# Rheological, Microstructural, and Physicochemical Characterization of Pasta Fortified with Carrot Pomace: A Comparative Study of Wheat Types and Carrot Varieties

**DOI:** 10.3390/foods15122201

**Published:** 2026-06-18

**Authors:** Marian Ilie Luca, Mădălina Ungureanu-Iuga, Viorela-Gabriela Ciobanu, Ana Batariuc, Silvia Mironeasa

**Affiliations:** 1 Faculty of Food Engineering, “Ştefan cel Mare” University of Suceava, 13th Universitatii Street, 720229 Suceava, Romania; marian.luca@usm.ro (M.I.L.); silviam@fia.usv.ro (S.M.); 2 Integrated Center for Research, Development and Innovation in Advanced Materials, Nanotechnologies, and Distributed Systems for Fabrication and Control (MANSiD), “Ştefan cel Mare” University of Suceava, 13th Universitatii Street, 720229 Suceava, Romania; gabriela.ciobanu@usm.ro; 3 Mountain Economy Center (CE-MONT), “Costin C. Kiriţescu” National Institute of Economic Researches (INCE), Romanian Academy, 050711 București, Romania; 4 Sanitary, Veterinary and Food Safety Directorate of Suceava, 2nd Scurta Street, 720223 Suceava, Romania; ana.batariuc@usm.ro

**Keywords:** by-product valorization, carrot waste, functional foods, nutritional profile, dough rheology

## Abstract

This study aimed to investigate the effects of incorporating carrot pomace from different varieties (Baltimore, Belgrado, Niagara, and Sirkana) into pasta formulations made from durum and common wheat flours, as well as to optimize the addition level and characterize the resulting products. To this end, dough rheological properties, pasta chemical composition, cooking behavior, color, texture, sensory attributes, and microstructure were evaluated. Increasing levels of carrot pomace significantly influenced flour functionality, dough rheology, pasta texture, cooking behavior, and color characteristics. Higher pomace addition resulted in increased flour water absorption, dough complex modulus and hardness, pasta fracturability, cooking losses, and contents of crude fiber and total yellow pigments, while reducing dough deformation resistance, pasta color intensity, and chewiness. The magnitude of these changes was dependent on the carrot variety used. Process optimization allowed the determination of variety-specific optimal inclusion levels of carrot pomace for both flour types. For durum wheat flour, optimal levels ranged from 6.34% to 9.25%, while for common wheat flour they ranged from 8.12% to 11.17%. At these levels, cooking losses remained within acceptable limits (<8%), yellow coloration was enhanced, and dough structure rigidity increased, accompanied by delayed starch gelatinization. Pasta samples containing Niagara and Sirkana pomace showed the highest contents of dietary fiber and yellow pigments, reflecting their elevated β-carotene levels. Sensory evaluation indicated improved overall acceptability compared with control samples. These results demonstrate the potential of carrot pomace as a functional ingredient for the development of nutritionally enriched, value-added pasta products.

## 1. Introduction

The valorization of by-products from the food and beverage industry, such as carrot pomace, is crucial because these materials are rich sources of fiber and bioactive compounds. Waste resulting from food processing, generally inexpensive, represents a loss of valuable biomass and nutrients and generates significant environmental and economic problems if improperly managed [1]. The consumption of pasta is increasing globally, and it is considered an excellent food matrix for the incorporation of bioactive substances in order to diversify the assortment range [2]. By incorporating these by-products into new food products, especially cereal-based products, the nutritional value of the products can be significantly improved. This is in line with the growing consumer demand for healthy and sustainable diets [1]. For example, studies have shown that high fiber intake can contribute to reducing the incidence of chronic disorders and diseases such as obesity, diabetes, cardiovascular disease, and cancer [3].

Carrot pomace is the by-product of the carrot juice extraction process and is a valuable source of total fiber, both insoluble and soluble [3,4]. Hernández-Ortega et al. [5] reported a fiber content of over 60% in carrot pomace, of which 79% was insoluble fiber. Carrot pomace contains significant amounts of carotenoids, mainly α- and β-carotenes, which are responsible for the color in orange varieties [6]. In addition, it also contains proteins, small amounts of lipids and minerals, such as Na, K, P, Ca, Mg, Cu, Mn, and Fe [3,7,8]. Carrots are rich in polyphenols and compounds with antioxidant activity, as well as natural sugars [8], some of which remain in the pomace after processing. In our previous study, we demonstrated that carrot pomace variety significantly influenced its physicochemical and functional properties, including color parameters, particle size distribution, hydration behavior, and microstructure. Furthermore, processing conditions were shown to affect the stability and retention of bioactive compounds, particularly carotenoids and phenolic compounds, which are important for the nutritional and technological quality of carrot pomace powders [7].

The addition of fiber-rich ingredients such as carrot pomace causes changes in the structural properties of wheat flour dough and higher viscoelasticity. It has been shown that the addition of fruit by-products causes a stiffening of the dough due to the high water absorption capacity of the fibers [9]. The incorporation of fruit or vegetable pomace can lead to increased resistance to deformation and extension [1,10]. Fibers compete with gluten for water, which can delay the development and hydration of the gluten network [11]. Another study demonstrated that insoluble fibers could disrupt the continuity of the gluten network, which causes a reduction in its structural strength [12]. Fibers can inhibit or change the type of intermolecular disulfide bonds in dough [13]. Carrot pomace addition may positively influence the shelf life and processing stability of pasta products due to its high dietary fiber, carotenoid, and phenolic compound content, which can improve water binding capacity and provide antioxidant protection against oxidative deterioration [12,14]. The high fiber content and water-binding capacity of vegetable pomaces may influence moisture redistribution and textural stability in pasta systems during storage by modifying the integrity of the starch–protein matrix [12,15]. In addition, the antioxidant compounds naturally present in carrot pomace may contribute to improved color stability and delayed lipid oxidation during storage [14].

The impact of the addition of fruit and vegetable pomace on the quality of bakery products and pasta depends on the addition dose and their chemical composition. An increase in the hardness of pasta was observed with the addition of carrot pomace [4]. Some studies have shown a decrease in cooking time and an increase in solids loss with increasing doses of vegetable by-products, such as broccoli leaf powder or onion peel, due to the weakening of the gluten matrix, which allows water to solubilize starch granules [2,16]. The addition of fiber-rich ingredients, such as pomace, can increase the water absorption capacity of pasta due to the ability of fibers to bind water molecules [12]. Carrot pomace improves the appearance of pasta by giving it an attractive color [4]. It has been stated that the decrease in the brightness of pasta with the addition of fruit or vegetable by-products is often perceived positively by consumers, who associate it with products rich in fiber [12]. Various fruit and vegetable by-products have been investigated as functional ingredients in pasta formulations due to their high dietary fiber, antioxidant, and bioactive compound content. Apple pomace, grape pomace, tomato pomace, beetroot pomace, and carrot pomace have been incorporated into pasta products to improve nutritional value while promoting the sustainable utilization of food processing residues [17,18,19,20,21]. Previous studies generally reported increases in dietary fiber, total phenolic compounds, antioxidant activity, and natural pigmentation in enriched pasta products, although high incorporation levels frequently impaired dough rheology, cooking quality, texture, and sensory acceptability [14,18,22].

The aim of this paper was to investigate the effects of carrot pomace dose and type of wheat flour with different protein content on dough and pasta characteristics, as well as to optimize the dose added for each carrot pomace variety and flour type. For this, the hydration capacity of flour, rheological properties of dough and pasta cooking behavior, chemical composition, texture, color, microstructure, and sensory properties were studied. While previous studies have reported the use of vegetable by-products, including carrot pomace, as functional ingredients in pasta, most have focused on a single carrot source, fixed inclusion levels, or a limited set of quality parameters. The present study advances existing knowledge by providing a comparative, variety-specific evaluation of carrot pomace incorporated into both durum wheat and common wheat pasta systems. A key novelty lies in the process optimization approach, which established distinct optimal addition levels for each carrot variety and flour type, ensuring nutritional enhancement without exceeding technological and sensory acceptability thresholds. Furthermore, this work offers a comprehensive, integrated assessment linking flour hydration behavior, dough rheology, starch gelatinization delay, pasta cooking performance, texture, color, and sensory attributes to the chemical composition of individual carrot varieties, particularly β-carotene and fiber content. The identification of cultivar-dependent effects on dough viscoelasticity, pigment retention, and fiber enrichment provides new insights not previously addressed in the literature. The comparative evaluation of carrot varieties and flour types is the main contribution of this paper.

## 2. Materials and Methods

### 2.1. Materials

Four carrot varieties (Baltimore, Belgrado, Niagara, and Sirkana) were obtained from a farmer in Bacău, Romania. Carrot pomace was produced by juice extraction using a centrifugal juicer Bosch MES3500 (BSH Hausgeräte GmbH, Munich, Germany) then dried in a hot-air convector ZRD-A5055 (Shanghai ZHICHENG Analytical Instruments Manufacturing Co., Ltd., Shanghai, China) at 60 °C for 24 h (0.5 cm layer). The dried pomace was ground and sieved to <200 µm. Wheat flour (durum or common) was replaced by carrot pomace in various amounts (3, 6, 9 or 12%).

Pasta dough was mixed for 10 min (300 g of composite flour) with gradual water addition to reach 45% moisture, rested for 15 min, and extruded into rigatoni (1.3 cm diameter) using a Kitchen aid mixer with pasta accessory (Whirlpool Corporation, Benton Harbor, MI, USA). Drying was performed in stages: room temperature (30 min), 40 °C (60 min), 80 °C (120 min), and 40 °C (120 min). The dried pasta was then cooled at room temperature for 12 h and kept at room temperature in polyethylene bags until analysis. Cooked pasta were freeze dried in a lyophilizer model BK-FD12 (Biobase, Jinan, China) before analysis.

### 2.2. Hydration Capacity

The hydration capacity of flour mixes was determined following the method of Bordei et al. [23], with modifications. A 2.5 g sample was mixed with 15 mL of water and stirred intermittently for 1 h. The suspension was centrifuged at 3000 rpm for 20 min, the supernatant removed, and the residue dried at 50 °C for 25 min. After cooling, the sample was weighed.

### 2.3. Proximate Composition and Mineral Content

The chemical properties (moisture, protein, lipid, ash) of lyophilized cooked pasta were analyzed using international methods (ICC): moisture (101/1), lipid (104/1), protein (105/2), and ash (105/1). The total amount of crude fiber was evaluated by acid and alkaline digestion using an automatic analyzer (Fibertec 2010, Tecator, Hillerod, Sweden). The carbohydrate content was calculated by difference, and, energetic value was established.

The determination of the mineral content of the raw materials and cooked and freeze-dried pasta was performed using a Shimadzu AAS-6300 atomic absorption spectrophotometer (air-acetylene), equipped with an ASC-6100 autosampler (Shimadzu Corporation, Kyoto, Japan). The mineral profile was established for four predominant elements: Zn, Na, Ca, Fe, and Cu. The samples were prepared by calcination at 550 °C (5 g of sample), according to the procedure described in the Shimadzu AAS manual. After cooling, 1 mL of 65% HNO_3_ was added, then the samples were transferred to 50 mL volumetric flasks and made up to volume with distilled water. The concentration of each element was expressed in mg/kg of sample. The coefficients of determination (*R*^2^) of the calibration curves ranged from 0.96 to 0.99.

### 2.4. Total Polyphenol Content (TPC)

Extracts were prepared following Ziobro et al. [24] by mixing 6 g of the sample (lyophilized cooked pasta) with 30 mL of 80% ethanol and shaking for 120 min. After centrifugation (4500 rpm, 15 min), the supernatant was used for phenolic analysis. Total phenolics were determined using the Folin–Ciocâlteu method, measuring absorbance at 750 nm. Results were expressed as mg gallic acid equivalents (GAE)/100 g sample.

### 2.5. Total Yellow Pigments (TYPs)

The total yellow pigment content of lyophilized cooked pasta was determined using a modified AACC International 14-50.01 method. An amount of 4 g of cooked, freeze-dried, and ground pasta was weighed, and 20 mL of water-saturated n-butanol was added [25]. The absorbance of the supernatant was measured with a spectrophotometer at a wavelength of 436 nm. The results obtained for each extracted sample were converted into yellow pigment concentration (mg/kg) using the extinction coefficient of β-carotene of 1.6632.

### 2.6. Rheological Behavior

The viscoelastic behavior of pasta dough during processing was evaluated using dynamic rheological tests performed in duplicate with a Thermo–HAAKE MARS 40 rheometer (Thermo Fisher Scientific Inc., Waltham, MA, USA) (parallel plates, 3 mm gap). Dough sheets (3 mm thick) were rested for 60 min and equilibrated at 20 °C for 120 s before testing. Measurements were conducted at 20 °C using Peltier temperature control, with Vaseline applied to prevent moisture loss. The linear viscoelastic region (LVR) was determined by stress sweep tests (by varying stress from 0.1 to 100 Pa, at 1 Hz frequency), and a stress of 15 Pa was selected for further analyses. Frequency sweep tests (varying frequency from 0.1 to 20 Hz) were performed in triplicate to determine the complex modulus (G*) and the storage (G′) and loss (G″) moduli. Thermal behavior was assessed by oscillatory tests at 1 Hz while heating from 20 to 100 °C at 4 °C/min, recording G′ and G″. Dough compliance was also evaluated using creep and recovery tests at 20 °C by applying a stress of 50 Pa for 60 s, followed by a 180 s recovery period.

### 2.7. Texture Analysis

All the texture measurements were made using a Perten TVT–6700 texturometer (Perten Instruments, Stockholm, Sweden).

#### 2.7.1. Dough Texture

To evaluate the texture of pasta dough, 50 g dough spheres were formed and then subjected to double compression to 50% of their original height using a 35 mm diameter cylindrical probe. During the test, a speed of 5.0 mm/s, a trigger force of 20 g, and a recovery time of 12 s between compressions were applied.

#### 2.7.2. Fracturability of Uncooked Pasta

The texture of the dried pasta was evaluated by applying a fracturability test. For this purpose, a breaking device with a sample clamping system adjusted to a width of 13 mm was used. Individual pieces of pasta were subjected to a flexural strength test, at a test speed of 3 mm/s and a trigger force of 50 g. The measurements were repeated at least 10 times.

#### 2.7.3. Cooked Pasta Texture

The determination of chewiness, elasticity, cohesiveness, gumminess, and firmness of cooked pasta was performed by applying a double compression of a single pasta piece of up to 75% of the initial height of the sample, using a cylindrical probe with a diameter of 35 mm, at a speed of 2.0 mm/s, a trigger force of 10 g and a recovery time of 12 s between compressions. At least 7 measurements were performed.

### 2.8. Cooking Behavior

The optimal cooking time of pasta was determined in triplicate, according to the method described by Espinosa-Solis et al. [26]. After a cooking time of 4 min, compressions of a piece of pasta between two glass plates were made every 30 s. The optimal cooking time (OCT) was recorded when no white starch particles were observed after compression.

To evaluate the solids loss during cooking (CL) and water absorption (WA), a quantity of 10 g of pasta was boiled in 20 mL of water, according to the OCT, and the resulting liquid was collected and dried at a temperature of 105 °C until a constant mass was obtained [27]. Subsequently, the resulting residue and the cooked pasta were weighed. This procedure was adopted to maintain consistency with a previously validated methodology for evaluating cooking properties of pasta formulations.

### 2.9. Color

Color parameters were measured in the CIE Lab system using a Konica Minolta CR-400 colorimeter, with at least three replicates per sample. Measured values included lightness (L*), red–green (a*), and yellow–blue (b*). Hue angle (H) was calculated from these parameters [28].

### 2.10. Microstructure and Surface Structure

For scanning electron microscopy (SEM) analysis, the cooked samples were cut into 2–3 mm thick cross-sections, mounted on carbon conductive tape, and sputter-coated with ~5 nm of gold. Images were acquired at an accelerating voltage of 20 kV, with representative micrographs taken at 500× magnification for all samples, using a Hitachi SU-70 Field Emission Scanning Electron Microscope (FE-SEM) (Hitachi High-Tech Corporation, Tokyo, Japan).

The surface structure and roughness of the dried pastas were analyzed using a Mahr CWM100 microscope (Mahr GmbH, Göttingen, Germany). For each sample, four different areas were observed, and the results were recorded accordingly. The images obtained were processed using Mountain Map software trial version (Digital Surf, Besançon, France), and the roughness was calculated as the mean of the computed profiles of 3 areas for each sample.

### 2.11. Sensory Properties

Cooked pasta was subjected to sensory analysis by a group of 15 semi-trained tasters, recruited from among the students and employees of the Faculty of Food Engineering at “Ștefan cel Mare” University in Suceava, Romania. The panelists were instructed to evaluate sensory attributes such as appearance, color, taste, odor, texture, and overall acceptability, using a 9-point hedonic scale. The coded samples were served to the sensory panel in random order and were rated on an intensity scale ranging from 1 to 9, where 1 means “I do not like it at all”, 5 means “I neither like it nor dislike it” and 9 means “I like it extremely much”. The sensory analysis was performed according to the Declaration of Helsinki and the protocol was approved by the Ethics Committee of the Stefan cel Mare University of Suceava (approval number 254/03.06.2025). Written informed consent was obtained from the panelists before testing.

### 2.12. Statistics and Experimental Design

All measurements were performed at least in duplicate. Statistical analysis was conducted using XLSTAT (Excel 2021 & 2024). A one-way ANOVA with Tukey’s test (*p* < 0.05) was applied to assess significant differences.

A D-optimal experimental design (Design Expert, trial version) was used to study the effects of two numerical factors—flour type-protein content (11% common wheat, 14% durum wheat) and carrot pomace dose (3, 6, 9, 12%), and one categorical factor (carrot variety: Baltimore, Belgrado, Niagara, Sirkana) on quality parameters of the flour, dough, and pasta. Responses included: hydration capacity (HC), dough complex modulus (G*), maximum compliance (Jmax), dough hardness, uncooked pasta Chroma, solid loss during cooking (CL), dry pasta fracturability, cooked pasta chewiness, total yellow pigments (TYPs), and crude fiber content. The factor “flour type-protein content” refers to the type of flour used (common wheat flour or durum wheat flour), which differs mainly in protein content, but also in other compositional and technological characteristics. All the responses were in triplicate, and a total of 96 runs were performed.

Predictive models were validated via multiple regression, ANOVA, Fisher test (F), significance (*p*), *R*^2^, and adjusted *R*^2^, predicted *R*^2^ and coefficient of variation (*CV*). Optimization of carrot pomace addition aimed to maximize HC, G*, Jmax, dough hardness, fracturability, Chroma, TYPs, and crude fiber content, while minimizing CL and chewiness. Dough hardness was maximized to ensure adequate dough consistency and mechanical strength during mixing and extrusion. Sufficient dough hardness contributes to maintaining structural integrity throughout pasta processing [29]. Jmax was included because it reflects the maximum deformation of the dough under an applied stress during creep testing. Within the experimental domain investigated, higher Jmax values were considered desirable because they indicate greater dough deformability, which may facilitate dough handling and extrusion [30]. Chewiness was minimized to avoid an excessively tough texture in the cooked pasta. Since the incorporation of dietary fiber-rich ingredients, such as carrot pomace, may increase textural toughness [31], reducing chewiness was considered desirable to improve eating quality and consumer acceptability.

Predicted data were validated by performing the experiments using the optimal parameters obtained. Optimal experimental and predicted conditions were validated using Student’s *t*-test.

## 3. Results and Discussions

### 3.1. The Influence of the Type of Flour, the Addition Level, and the Type of Carrot Pomace on the Properties of Wheat Flour, Dough, and Pasta

The matrix of experimental data used for mathematical modeling is presented in Table S1 in the Supplementary Materials. Significant differences (*p* < 0.05) were observed in the characteristics of flours, doughs, and pastas depending on the protein content of the flour (its type) and the dose of carrot pomace, while the carrot variety had a lesser influence.

The experimental data for all ten responses were modeled using the 2FI type mathematical model (Table 1), which proved to be suitable to explain the variation in the data (0.75 < *R*^2^ < 0.98). The three factors, flour type-protein content (A), addition dose (B), and carrot variety (C), and their interactions exerted a significant influence (*p* < 0.05) on hydration capacity (HC), complex modulus (G*), maximum compliance (Jmax), Chroma parameter, total yellow pigment content, and fiber content. The interaction between the addition dose and carrot variety had no significant influence (*p* > 0.05) on dough hardness, CL, and fracturability of flour pastas. The interaction between flour type-protein content, and addition dose did not show a significant effect (*p* > 0.05) on CL, fracturability, and chewiness of pasta. Chewiness was not significantly influenced (*p* > 0.05) by flour type-protein content (Table 1), but it depends on the dose and variety of carrot pomace. All three factors have a significant effect (*p* < 0.05) on dough hardness, solids loss on boiling, and fracturability of pasta.

The HC increased at higher addition doses (Figure S1, Supplementary Materials), and the degree of change was dependent on the carrot pomace variety, with the greatest changes observed for Baltimore and Sirkana varieties. Ahmad et al. [32] reported an increase in the water absorption capacity of wheat flour due to the fiber content of carrot pomace, as there is an increase in the competition for water between pomace and flour. Studies have reported that the incorporation of fiber into wheat flour dough increases water absorption due to the higher water affinity of pomace powder compared with wheat flour: the hydroxyl groups of pomace fibers can generate hydrogen bonds with water [33]. The differences between flour mixes with different carrot pomace varieties could be due to their chemical composition [8]. The functional properties of the flour mixtures used in this study, including water retention and swelling capacities, were previously characterized and reported in our previous paper [34]. Durum wheat flour with higher protein content determined higher values for HC. Durum wheat flour has been stated to have a higher water-binding capacity due to its protein content [35]. Other studies have also reported a higher HC of durum wheat flour compared with common wheat flour [36,37]. The predictive models obtained for the hydration capacity, corresponding to each carrot pomace variety, are presented by the following relationships (Equations (1)–(4)):

(1) $${HC}_{Baltimore}\left(\%\right)=67.02-0.98A-1.40B+0.35AB$$

(2) $${HC}_{Belgrado}\left(\%\right)=24.74+2.64A-2.49B+0.35AB$$

(3) $${HC}_{Niagara}\left(\%\right)=-27.68+6.42A-1.88B+0.35AB$$

(4) $${HC}_{Sirkana}\left(\%\right)=-20.96+5.82A-1.28B+0.35AB$$

Increasing the dose of carrot pomace addition led to an increase in the complex modulus (G*), and the changes were more pronounced in the samples with Baltimore and Sirkana varieties (Figure S2, Supplementary Materials), probably due to the different chemical composition between the varieties [7]. Similar to these results, Ahmad et al. [32] observed that with increasing the dose of carrot pomace, wheat flour dough becomes harder and less extensible and thus presents higher values for viscoelastic moduli due to the reduction in gluten proteins. In another study, compared with the control sample, the addition of 10% berry pomace led to stiffening of the dough (higher values for G*), probably due to the bonds formed between the pomace compounds and wheat proteins, as well as the decrease in the amount of free water available in the dough [38]. Alba et al. [11] evaluated the rheology of wheat flour dough with blackcurrant pomace and insoluble and soluble fibers extracted from it (5–20%) and suggested that the stiffening of dough with insoluble fibers was due to the increased cellulose content, and the effect was more pronounced at higher doses. Dough made from common wheat flour, with a lower protein content, showed lower values of the complex modulus (G*) compared with that made from durum wheat flour. It has been shown that dough made from durum wheat flour has higher strength, stiffness, and tenacity than that made from common wheat [39]. These results could be due to the type of gluten proteins and/or the different hydration levels of the dough between the two types of wheat flours [40]. In our earlier work, carrot pomace incorporation resulted in a dose-dependent decrease in wet gluten content and gluten deformation index, suggesting a dilution of gluten-forming components due to the replacement of wheat flour with a non-gluten ingredient [34]. Moreover, the high fiber content of carrot pomace may interfere with gluten network development by competing for available water and limiting protein hydration. These effects can lead to a less cohesive and less continuous gluten matrix, accompanied by modifications in water absorption, retention, and dough organization, which are considered the primary mechanisms governing the behavior of the pasta systems investigated in the present study [34]. Equations (5)–(8) show the parameters of the predictive model for the complex modulus, corresponding to each carrot pomace variety.

(5) $${G*}_{Baltimore}\left(Pa\right)=-1.05\cdot 10^{6}+0.96\cdot 10^{5}A+1.20\cdot 10^{5}B-0.05\cdot 10^{5}AB$$

(6) $${G*}_{Belgrado}\left(Pa\right)=-3.99\cdot 10^{5}+0.50\cdot 10^{5}A+0.85\cdot 10^{5}B-0.05\cdot 10^{5}AB$$

(7) $${G*}_{Niagara}\left(Pa\right)=-9.08\cdot 10^{5}+0.86\cdot 10^{5}A+1.01\cdot 10^{5}B-0.05\cdot 10^{5}AB$$

(8) $${G*}_{Sirkana}\left(Pa\right)=-3.71\cdot 10^{5}+0.31\cdot 10^{5}A+1.22\cdot 10^{5}B-0.05\cdot 10^{5}AB$$

The maximum compliance recorded during creep and recovery testing (Jmax) showed a decreasing trend with increasing carrot pomace dosage, the most pronounced trend being observed in the case of the Belgrado variety (Figure S3, Supplementary Materials), probably due to its chemical composition [7]. Mironeasa and Codină [41] observed an increase in the compliance of wheat flour dough with the addition of tomato pomace greater than 10% and attributed this behavior to the decrease in gluten content. The mechanism of the decrease in the Jmax parameter is associated with the reduction in free water available in the dough and the strengthening of the starch protein network due to carrot pomace components [11,38,42]. The samples from common wheat flour, with a lower protein content, showed lower values compared with those from durum wheat, indicating a lower resistance to deformation. These results were in agreement with those obtained by Edwards et al. [43] who found that doughs from durum wheat flour showed lower creep flexibility and a more resistant structure to deformation compared with those from common wheat. This is due to the molecular structure of gluten proteins, their mobility, and the number of physical bonds in the glutenin fraction [43]. Equations (9)–(12) show the predictive model for maximum compliance (Jmax) for each carrot pomace variety.

(9) $${J_{max}}_{Baltimore}\left(Pa\cdot s\right)=23.20-1.33A-2.22B+0.15AB$$

(10) $${J_{max}}_{Belgrado}\left(Pa\cdot s\right)=39.46-2.39A-2.52B+0.15AB$$

(11) $${J_{max}}_{Niagara}\left(Pa\cdot s\right)=24.39-1.52A-2.18B+0.15AB$$

(12) $${J_{max}}_{Sirkana}\left(Pa\cdot s\right)=26.35-1.48A-2.40B+0.15AB$$

Dough hardness increased with increasing addition dose for all carrot pomace varieties (Figure S4, Supplementary Materials). Increasing the dose of carrot pomace causes higher water absorption by the fibers, which leads to the progressive dehydration of gluten and starch, resulting in a firmer dough [44]. The fibers in the pomace interact with the dough matrix and gluten development by modifying the rheological properties, reducing gluten yield and extensibility, disrupting gluten structure, interfering with its formation, and limiting water availability, which leads to increased dough firmness [1]. Nour et al. [45] reported an increase in the hardness of biscuit dough with increasing doses of blackcurrant and blueberry powder. Durum wheat flour with a higher protein content led to higher dough hardness values compared with common wheat flour. These results can be attributed to differences between wheat genotypes [46], respectively to the higher protein content of durum wheat flour [40]. The structure of proteins, respectively their molecular mass and its distribution in glutenin fractions, play an important role in dough hardness [43]. The predictive models for dough hardness, related to each carrot pomace variety, are expressed by Equations (13)–(16).

(13) $${Dough\;hardness}_{Baltimore}\left(N\right)=-36.75+4.57A+4.14B-0.19AB$$

(14) $${Dough\;hardness}_{Belgrado}\left(N\right)=-49.18+5.38A+4.05B-0.19AB$$

(15) $${Dough\;hardness}_{Niagara}\left(N\right)=-68.41+7.16A+4.12B-0.19AB$$

(16) $${Dough\;hardness}_{Sirkana}\left(N\right)=-41.22+4.71A+4.01B-0.19AB$$

The color of uncooked pasta was significantly (*p* < 0.05) influenced by the dose, carrot pomace variety, and flour type. Increasing the addition dose resulted in an increase in the color intensity described by the Chroma parameter, and the trend was more pronounced in the case of Baltimore and Belgrado varieties (Figure S5, Supplementary Materials). The change in pasta color is due to the pigments in carrot pomace that differ depending on the variety [47,48]. Typically, orange carrots have a significant content of β-carotene and α-carotene, but also contain lutein and zeaxanthin, while red carrots also have lycopene, in addition to the amount of α-/β-carotene and lutein [6]. Carotenoids are sensitive to temperature, light, and oxygen, and during the drying of pasta, their isomerization and oxidative reactions can lead to thermal degradation, thus reducing the color attributes, sensory properties, and nutritional value of the product [49]. Pasta made from common wheat showed lower values of Chroma compared with those made from durum wheat, which has a higher protein content. Carotenoids provide the yellow color of the endosperm of durum wheat and, consequently, of the flour, while in common wheat flour, their content is lower [50,51]. Equations (17)–(20) show the predictive models for the variation of the Chroma parameter depending on the type of flour—protein content, dose, and carrot pomace variety.

(17) $${Chroma}_{Baltimore}=6.89+1.37A+0.49B-0.06AB$$

(18) $${Chroma}_{Belgrado}=3.27+1.62A+0.59B-0.06AB$$

(19) $${Chroma}_{Niagara}=16.45+0.55A+0.65B-0.06AB$$

(20) $${Chroma}_{Sirkana}=17.13+0.58A+0.65B-0.06AB$$

Losses of soluble solids (CL) during cooking are an essential characteristic of pasta quality [52]. As the addition dose increased, a more pronounced release of solids into the boiling water was observed regardless of the carrot pomace variety (Figure S6, Supplementary Materials). The weakening of the gluten matrix of pasta enriched with fiber-rich ingredients may cause a greater leaching of starch and other components into the boiling water, a fact also supported by the research carried out by Ta et al. [53] for pasta with the addition of mulberry pomace (5–20%). Drabinska et al. [16] demonstrated that the introduction of broccoli by-products (2.5–5%) into pasta also led to an increase in solids losses during cooking, and the values were below the 8% acceptability limit. In the present study, it was observed that at the dose of 12% carrot pomace, the acceptability limit for soluble solids losses was exceeded, depending on the variety. It has been shown that fiber-rich ingredients, such as carrot pomace, can inhibit the formation of the gluten matrix during the shaping process of flour pasta; thus, water penetrates more easily into their structure during boiling, and starch granules are released more easily [2,54]. The predictive model obtained for each carrot pomace variety described the variation in soluble solids losses (CL) depending on the type of flour—protein content, and addition dose (Equations (21)–(24)).

(21) $${CL}_{Baltimore}\left(\%\right)=-1.02+0.40A+0.26B+0.01AB$$

(22) $${CL}_{Belgrado}\left(\%\right)=-0.61+0.31A+0.32B+0.01AB$$

(23) $${CL}_{Niagara}\left(\%\right)=2.73+0.08A+0.36B+0.01AB$$

(24) $${CL}_{Sirkana}\left(\%\right)=-1.79+0.51A+0.21B+0.01AB$$

Fracturability is an important property that provides information on the resistance of pasta to handling and transportation and refers to the maximum force at which pasta breaks. Increasing the addition dose led to an increase in the force required to break the pasta, with the most pronounced upward trend observed for the Baltimore variety (Figure S7, Supplementary Materials). Another study demonstrated that biscuits prepared with unripe banana flour were more resistant to breakage than those in the control group due to a well-organized resistant starch and amylopectin matrix [55]. The formation of covalent and non-covalent bonds between gluten and carrot pomace polyphenols could positively influence the structure of the gluten network, according to the study by Wang et al. [56]. In addition, fiber-containing components naturally present in carrot pomace may promote the conversion of sulfhydryl groups (-SH) into disulfide bonds (-S-S-), thereby contributing to the formation of a more cohesive gluten network [57]. Durum wheat flour samples, with a higher protein content, showed higher fracturability compared with common wheat samples, which have a lower protein content. The superiority of durum wheat for pasta is not only due to its higher protein content, but especially due to the specific composition of the protein fractions, where those of low molecular weight glutenins (LMW-GS) in durum wheat form a dense network specific to pasta, in contrast to high molecular weight glutenins (HMW-GS) in common wheat which contribute to the formation of the weaker structure specific to bread [40]. The predictive models describe the variation in fracturability depending on the dose of carrot pomace and the type of flour—protein content is expressed by Equations (25)–(28).

(25) $${Fracturability}_{Baltimore}\left(N\right)=-9.90+3.09A+0.94B-0.15AB$$

(26) $${Fracturability}_{Belgrado}\left(N\right)=14.97+1.32A+0.90B-0.15AB$$

(27) $${Fracturability}_{Niagara}\left(N\right)=39.64-0.59A+0.29B-0.15AB$$

(28) $${Fracturability}_{Sirkana}\left(N\right)=78.35-4.41A+0.86B-0.15AB$$

Increasing the dose of carrot pomace caused a decrease in the chewiness of cooked pasta (Figure S8, Supplementary Materials), and the most pronounced trend was observed for the Belgrado and Sirkana varieties. Nguyen et al. [22] also reported a decrease in the chewiness of pasta with increasing the dose of cashew apple pomace, probably due to the disruption of the gluten network, which makes the pasta break more easily under the influence of external forces. Another study demonstrated that the addition of 15% apple pomace caused a decrease in the chewiness of noodles due to structural changes generated by the presence of fibers that absorb more water and influence starch gelatinization [58]. For carrot pomace samples from Baltimore and Belgrado varieties, chewiness was higher when durum wheat flour with a higher protein content was used compared with common wheat flour, while for Sirkana and Niagara varieties, the trend was the opposite. These results could be attributed to the different chemical composition between carrot varieties, which determines different interactions between dough components [7,47]. The predictive models obtained, corresponding to each carrot pomace variety, described the variation in chewiness depending on the carrot pomace dose and the type of flour—protein content is expressed by Equations (29)–(32).

(29) $${Chewiness}_{Baltimore}\left(N\right)=8.12+1.32A-0.32B-0.01AB$$

(30) $${Chewiness}_{Belgrado}\left(N\right)=5.19+1.93A-0.77B-0.01AB$$

(31) $${Chewiness}_{Niagara}\left(N\right)=42.08-1.42A-0.10B-0.01AB$$

(32) $${Chewiness}_{Sirkana}\left(N\right)=57.72-2.24A-0.54B-0.01AB$$

The content of total yellow pigments (TYPs) increased with increasing addition dose, the increase being more pronounced in the case of Sirkana and Niagara varieties (Figure S9, Supplementary Materials). Carrot pomace is rich in β-carotene, and its content varies depending on the variety [47]. The yellow color of carrot pomace is given by the presence of carotenoids, especially β-carotene, lutein, as well as polyphenols [59]. The predictive models that describe the variation in the content of total yellow pigments depending on the dose of carrot pomace and the type of wheat flour—protein content, corresponding to the carrot pomace variety, is expressed by Equations (33)–(36).

(33) $${TYP}_{Baltimore}\left(mg/kg\right)=-0.39+0.05A+0.08B-0.01AB$$

(34) $${TYP}_{Belgrado}\left(mg/kg\right)=-0.23+0.04A+0.08B-0.01AB$$

(35) $${TYP}_{Niagara}\left(mg/kg\right)=-0.01+0.02A+0.10B-0.01AB$$

(36) $${TYP}_{Sirkana}\left(mg/kg\right)=-0.19+0.03A+0.09B-0.01AB$$

Carrot pomace contributed to an increase in the crude fiber content of the pasta, as evidenced by the progressive rise in total crude fiber with increasing levels of carrot pomace incorporation (Figure S10, Supplementary Materials). Previous studies have shown that the carrot pomace from the four varieties used in the present study is rich in fiber [7]. Pasta with carrot pomace from the Sirkana variety presented the highest crude fiber content (Table S1), which is in accordance with the highest fiber content of this variety compared with the others [7]. Ta et al. [53] also reported an increase in total fiber content with increasing mulberry pomace dose. The variation in fiber content depended on the type of flour—protein content, and carrot pomace dose was described by the predictive models through Equations (37)–(40).

(37) $${Crude\;fiber}_{Baltimore}\left(\%\right)=-0.29+0.03A+0.20B-0.01AB$$

(38) $${Crude\;fiber}_{Belgrado}\left(\%\right)=0.02-0.01A+0.21B-0.01AB$$

(39) $${Crude\;fiber}_{Niagara}\left(\%\right)=-0.79+0.06A+0.21B-0.01AB$$

(40) $${Crude\;fiber}_{Sirkana}\left(\%\right)=0.61+0.01A+0.17B-0.01AB$$

### 3.2. Optimization of Flour Type and Carrot Pomace Dose

The optimal dose of carrot pomace from each variety and for each type of flour was established according to the criteria presented in Section 2.12. Thus, for durum wheat flour (F1) an optimal dose of 6.34% was established for the Baltimore variety (F1Ba), 9.25% for the Belgrado variety (F1Be), 8.30% for the Niagara variety (F1Ni), and 7.71% for the Sirkana variety (F1Si). In the case of common wheat flour, optimal doses of 8.12% were obtained for the Baltimore variety (F2Ba), 9.06% for the Belgrado variety (F2Be), 10.36% for the Niagara variety (F2Ni), and 11.17% for the Sirkana variety (F2Si) (Table 2).

In general, the differences between the experimental and predicted values were less than 10%, with a few exceptions that did not exceed 15% differences. The highest value for the hydration capacity of the flour (HC) was recorded for the F1Be sample (88.25%). Gluten proteins are the main water-binding compounds, so their type and quantity significantly influence HC [60]. In addition, the presence of fibers causes an increase in HC due to the binding of water by hydroxyl groups [61].

The highest value of the complex modulus (G*) (766.35 kPa) was observed for the F2Si sample from common wheat flour, which also has the highest carrot pomace content (11.17%). Glutenin is the protein component responsible for the elasticity of the dough, while gliadin influences the viscosity [62]. Fibers reduce the amount of water available for the gluten network, which leads to a decrease in deformability because the gluten formed is denser and more rigid [9]. Interactions between gluten proteins and dough fibers play a critical role in modifying protein structure, disulfide and hydrogen bonds, which lead to significant changes in dough rheological properties [63]. The maximum compliance (Jmax) ranged from 1.64 Pa·s (F2Si) to 5.40 Pa·s (F2Be), suggesting a higher resistance to dough deformation with 9.06% Belgrade (F2Be) compared with the other samples. The network characteristics of low molecular weight glutenins, such as network area, lacunarity, and number of junctions, play an essential role in dough elasticity, and therefore in its ability to deform [64]. The dough hardness ranged between 25.35 N (F2Ni) and 44.71 N (F1Ni) and was also influenced by the presence of carrot pomace fibers. The highest value of the Chrome of the pasta was observed for the F1Ni sample (24.91), and the lowest value for F1Be (20.03). The color of the final product depends on the amount of carotenes responsible for the yellow color in the carrot pomace, a quantity that was shown to be different depending on the variety [47]. The lowest losses of soluble solids upon boiling were obtained at higher addition doses, which was expected. Boiling solids losses (CL) ranged from 6.45% (F2Ba) to 7.94% (F1Si), values that fall within the acceptable limits [2]. The integration of granular particles into pasta dough can compromise structural integrity by inhibiting the homogeneous wetting of components and obstructing the formation of the gluten network [12], which leads to increased boiling solids losses.

The fracturability of pasta varied between 40.83 N (F1Si) and 60.48 N (F1Be), and the chewiness indicated values between 18.00 N (F2Ba) and 24.93 N (F2Si). The cooked pasta samples with the addition of carrot pomace from the Niagara and Sirkana varieties showed the highest content of total yellow pigments and crude fibers (0.51 mg/kg and 1.26% for F2Ni, respectively 0.52 mg/kg and 1.66% for F2Si), which can be correlated with the high β-carotene and fiber content of these varieties [47].

### 3.3. Characterization of the Optimal Samples

#### 3.3.1. Rheological Properties of Dough

**Figure 1:**
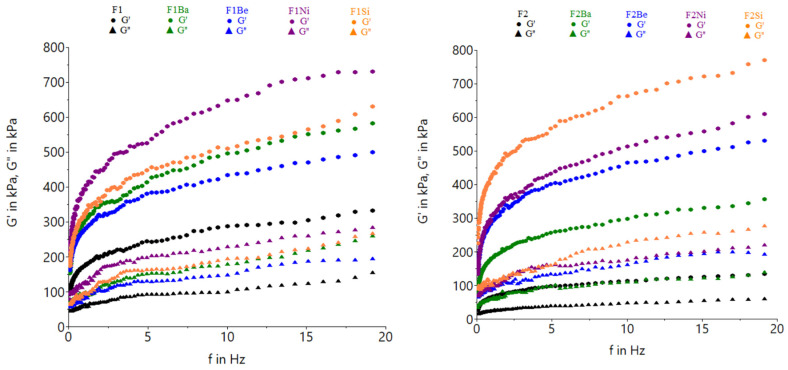
Variation in viscoelastic moduli with frequency.

The variation in the elastic moduli (G′) and viscosity (G″) with frequency during oscillatory testing is shown in Figure 1. All tested samples had a behavior characteristic of solids since G′ > G″. Dough samples containing carrot pomace showed higher viscoelastic moduli compared with the control samples, indicating an increase in the rigidity of the gluten network.

The highest values of G′ and G″ moduli were obtained for samples with carrot pomace from Niagara and Sirkana varieties in common wheat flour, F2Ni and F2Si, which were formulated with the highest addition doses. Salari et al. [65] also reported an increase in G′ and G″ moduli with the addition of carrot or apple pomace in the dough for biscuits made from wheat flour. The increase in the viscoelastic moduli may be related to the incorporation of polyphenol- and fiber-rich carrot pomace. Literature reports suggest that polyphenol–protein interactions and the presence of fibrous particles can influence dough structure and rheological behavior. However, as these interactions were not directly evaluated in the present study, they should be regarded as possible contributing factors rather than confirmed mechanisms [66]. Kaur et al. [67] observed an increase in G′ and G″ values in pasta dough with the addition of orange or cucumber peel powder. It was observed that the hydration level, air retention capacity, and the ability of the components to bind water significantly influence the rheological properties of the dough, and the values of G′ and G″ moduli increase considerably when components with high water absorption, such as fibers, are used, which determines a low degree of dough hydration [67]. In the case of durum wheat flour, higher values for G′ and G″ were obtained compared with those corresponding to dough made from common wheat flour. The rheological properties of dough depend mainly on the quality and quantity of gluten, which varies depending on the wheat variety, with quality differences being correlated with the glutenin/gliadin ratio and glutenin quality, factors that determine the ratio between the elastic and viscous components of the dough [68]. The greater stability of dough made from durum wheat flour compared with common wheat flour may be due to the higher ratio of low-molecular-weight glutenins to high-molecular-weight ones. In the presence of an excess of low-molecular-weight glutenins, other such glutenins can replace those that detach, constantly filling the gaps in the gluten network and thus enhancing its stability during kneading [69].

**Figure 2:**
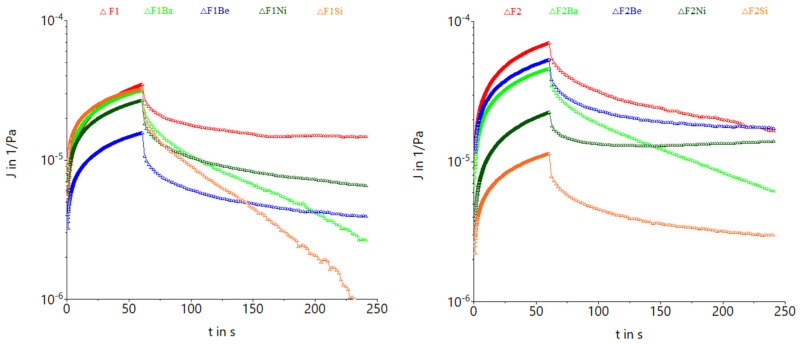
Creep and recovery curves.

Creep and recovery testing revealed differences between dough samples depending on the type of flour, the variety, and the amount of carrot pomace added. The addition of carrot pomace caused a decrease in compliance values compared with the control samples. The dough made from common wheat flour showed higher compliance compared with that made from durum wheat flour. Higher doses of pomace (F1Be, F2Ni, and F2Si) led to the most pronounced decreases in the creep and recovery curves (Figure 2), which indicates a higher resistance to deformation of the dough. For durum wheat dough samples (Figure 1), the addition of carrot pomace improved the recovery behavior compared with the control sample (F1), as evidenced by the more pronounced decrease in compliance during the recovery phase. Samples F1Si and F1Ba showed the highest recovery capacity, indicating a more elastic structure and lower permanent deformation after stress removal, whereas the control sample exhibited the weakest recovery behavior and a more viscous response. In the case of common wheat dough samples (Figure 2), the recovery strain was also affected by carrot pomace incorporation, but the effect depended strongly on the pomace variety. Sample F2Si exhibited the greatest recovery and the lowest compliance values during the recovery stage, suggesting enhanced elasticity and structural stability. Conversely, the control sample (F2) and F2Be showed weaker recovery behavior and higher residual deformation, indicating a more pronounced viscous character.

High values of maximum creep compliance could indicate a high moisture content in the gluten matrix, and the increased ability of fibers to retain water molecules through hydrogen bonds accelerates the competition for water between proteins, starch, and fibers, reducing water distribution and lubrication of the dough matrix [70]. Dokić et al. [71] observed that the values of creep and recovery compliance curves for samples with oat, potato, and pea fibers decreased compared with the control sample, indicating a stiffer and less elastic dough structure. Ranasinghe et al. [70] reported that dough samples with aqueous extracts of date seeds showed lower compliance values compared with the control sample due to polyphenols that induce depolymerization reactions and intermolecular interactions, leading to a more compact and ordered gluten network with increased resistance to deformation. The extensibility of wheat flour dough is influenced by gluten development, which is influenced by non-starch polysaccharides, such as soluble fibers, through non-covalent interactions and limiting water availability, which leads to a more rigid gluten network, with reduced water mobility and increased resistance to deformation [72]. It has been shown that molecular weight significantly influences relaxation time; the lower the molecular weight, the shorter the relaxation time, and a narrower molecular weight distribution leads to a more pronounced decrease in relaxation modulus, which differentiates the creep and recovery behavior of wheat flour doughs depending on the gluten quality [68].

**Figure 3:**
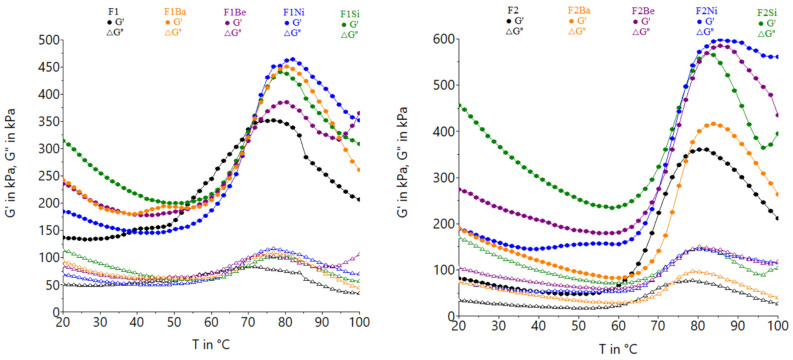
Variation in viscoelastic moduli with temperature.

The rheological behavior during heating of pasta dough can provide information about the changes that occur during cooking (Figure 3). The addition of carrot pomace led to an increase in the G′ and G″ moduli during heating compared with the control samples, depending on the dose. In addition, a delay in starch gelatinization can be observed as the maximum value of the G′ modulus was reached at higher temperatures compared with the control samples. Similarly, Reißner et al. [38] reported higher values for the complex modulus (G*) in the case of dough with blackcurrant pomace compared with the control sample.

The results were consistent with those obtained by Schmidt [73] for bagel dough with the addition of blackcurrant pomace due to interactions between the pomace fibers and flour proteins that can disrupt the starch–protein matrix. A strengthening of the dough structure was observed at temperatures above 80 °C, which is suggested by the increase in moduli as a result of starch gelatinization, protein denaturation, and moisture evaporation [73]. Similar to the present research, Hashemi et al. [74] reported a delay in starch gelatinization with the addition of goji powder; since gelatinization occurs through the absorption of water by starch molecules, the presence of fibers and their competition for water could possibly delay the absorption of water by starch and, implicitly, the gelatinization process. Compared with the common wheat flour dough, the durum wheat dough presented lower values of the G′ and G″. These differences between the common wheat and durum wheat dough can be attributed to the protein content and different interaction mechanisms between starch and protein, according to the observations of Singh et al. [75] who reported correlations between viscoelastic moduli and total protein content.

#### 3.3.2. Chemical Composition and Bioactive Compounds

The addition of carrot pomace significantly influenced the nutritional value of cooked pasta compared with the control sample (Table 3). The results obtained show a decrease in protein content due to the dilution effect induced by carrot pomace, which is rich in fiber [7].

An improvement in mineral (ash) and fiber content was observed compared with the control samples, while the content of carbohydrates and total polyphenols (TPC) decreased depending on the variety and amount of carrot pomace added. The energy value of the pasta ranged from 351.61 kcal/100 g (F2Ni) to 358.94 kcal/100 g (F2), and compared with the control samples, the values decreased with the addition of carrot pomace. Similarly, Ta et al. [53] reported a decrease in protein content and an increase in the amount of crude fiber and ash in pasta with the addition of mulberry pomace. The loss of soluble substances during boiling may explain the irregular variation in lipid content and the decrease in TPC compared with the control samples, mainly due to the differences in structure caused by the carrot pomace varieties with distinct chemical composition and the amount added, similar to previous observations reported by Gumul et al. [76]. Gałkowska et al. [77] also reported a decrease in the carbohydrate content of pasta with the addition of blackcurrants compared with the control sample, mainly due to the presence of fibers and other non-carbohydrate components present in the pomace. After boiling, the loss of bound phenolic compounds occurs [78]. The interaction of proteins and carbohydrates with polyphenols leads to the formation of covalent bonds, and these complexes can reduce the extractability of polyphenols in analytical extracts, resulting in apparently lower TPC values [79].

**Figure 4:**
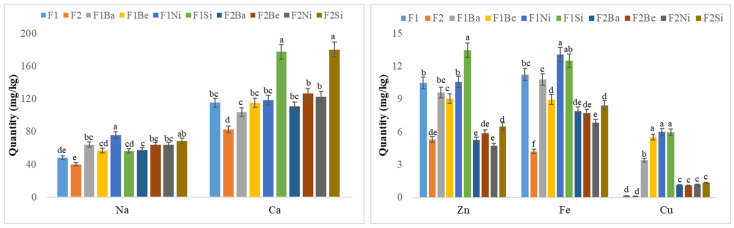
Mineral content of the optimal pasta: a–f—distinct letters indicate significant differences between samples (*p* < 0.05).

The mineral content of cooked pasta is represented in Figure 4. Compared with the control samples, an increase in the content of Na, Ca, Zn, Fe, and Cu is observed, with a few exceptions. The improvement in mineral content can be observed, especially in the case of samples with a higher addition of carrot pomace (F2Ni, F2Si), due to its contribution. The lower values for Ca, Zn, and Fe in the case of samples F1Ba and F1Be compared with the control sample (F1) could be attributed to the chemical composition of carrot pomace, which determines different interactions with the dough components and which, ultimately, can lead to mineral losses during boiling due to the weakening of the gluten matrix [12]. According to the data reported by Wang et al. [80], the mineral content increased in pasta with the addition of spinach and cabbage pomace, but boiling led to its decrease, possibly due to the loss of soluble compounds. Gull et al. [81] stated that pasta enriched with millet had a content of 4.23 mg Ca, 3.99 mg Fe and 1.68 mg Zn. In the present study, the values were higher due to the rich mineral content of the carrot pomace used. Durum wheat flour was found to be richer in minerals compared with common wheat flour. The average Cd, Cu, Fe, and Mo contents of common wheat varieties were lower compared with those of durum wheat, according to research conducted by Harmankaya et al. [82], which supports the results of the present study.

#### 3.3.3. Physical and Structural Properties

The addition of carrot pomace caused changes in the parameters corresponding to the boiling behavior depending on the dose. A slight decrease in the OCT of pasta was observed with the addition of carrot pomace compared with the control samples (Table 4).

The increase in WA recorded higher values with the addition of carrot pomace in durum wheat flour compared with the control sample, while for pasta made from common wheat flour, an opposite trend was observed. Common wheat flour presented higher values for OCT and WA compared with durum wheat flour. The observed OCT changes may be caused by the disruption of the gluten network by the addition of carrot pomace rich in fiber, which favored water absorption and contributed to shortening the boiling time. Similar results were obtained by Michalak-Majewska et al. [2] for pasta with onion peel powder. Studies have shown that the incorporation of wheat germ and bran particles disrupts the gluten network, favoring water retention in pasta and thus determining a shorter optimal cooking time compared with conventional pasta [83]. According to the data presented by Makhlouf et al. [84], durum wheat pasta enriched with fiber showed a greater volume increase during cooking compared with the control sample, an observation that can be attributed to the high water-retaining capacity of fibers. While the addition of barley resulted in a trend of increasing water absorption with increasing enrichment level, the oat bran samples showed an inverse relationship, with water absorption decreasing as the amount increased [84]. The high level of starch gelatinization and the disruption of the gluten–starch network are the main reasons for the increased water absorption of pasta [83]. On the other hand, Yousif et al. [85] demonstrated that water absorption in pasta with added protein occurs due to the competition between the added protein and starch for water. Thus, the differences between durum wheat and common wheat flour pastas in terms of volume growth could be attributed to the differences in structure and protein content between the flours.

The solids loss on boiling (CL) was significantly higher in the case of pasta with carrot pomace added compared with the control samples. The results were in line with those obtained by other researchers for pasta with chicory or turmeric by-products added [86,87] which showed higher solids loss compared with the control sample due to a weaker and less cohesive structure, which could favor the release of soluble compounds.

The color parameters of the pastas change both as a result of the addition of carrot pomace and during the cooking process (Table 4). Compared with the control samples, the lightness (L*) decreased in the carrot pomace samples, while the yellowness (b*) increased. The red hue (a*) increased in durum wheat flour and decreased in common wheat pasta. The hue index value (H) indicates a pale-yellow color of carrot pomace flour pasta, a value that generally decreases after cooking. Following heat treatment, an intensification of the yellow (increased b*) and red (increased a*) hues of the carrot pomace flour pasta was observed. On the other hand, durum wheat flour pasta intensified its green hue (decreased a*) and diminished its yellow color (decreased b*) after cooking, while durum wheat flour pasta became less reddish (decreased a*) and more yellow (increased b*).

Although durum wheat flour had a higher protein content, pasta color is influenced by carotenoid pigment concentration and pigment stability. Durum wheat semolina is naturally richer in yellow carotenoids, particularly lutein, which contribute to higher b* and H values and the characteristic bright yellow appearance of pasta products [88]. Conversely, common wheat flour generally contains lower carotenoid levels and may exhibit higher polyphenol oxidase activity, promoting pigment degradation and darker color formation during processing [50,89]. In durum wheat, lutein and zeaxanthin, as representatives of carotenoids, are the main components contributing to the color of flour and pasta [88]. Giannone et al. [90] stated that the yellow hue is appreciated by pasta consumers. According to a study, the enrichment of durum wheat pasta with freeze-dried carrot pomace capsules resulted in a significant increase in the yellow index, due to the presence of α-carotene, β-carotene, and cis-β-carotene [88]. An increase in the intensity of the yellow hue was also observed after boiling, similar to the results of the present study, an increase explained by the transformation of pigments during cooking [81,88].

Another study showed that the color parameters of pasta made from common wheat flour showed higher values in pasta with the addition of carrot flour due to the orange color of this ingredient [91]. In pasta, the yellow color is naturally present when using durum wheat flour (*T. durum*) due to the presence of pigments, which does not happen in the case of common wheat flour (*T. aestivum* L.) [91]. It has been shown that the heat treatment of carrot products can produce color changes due to the cis-isomerization of α- and β-carotenoids [92]. This could explain the color change in pasta after cooking.

**Figure 5:**
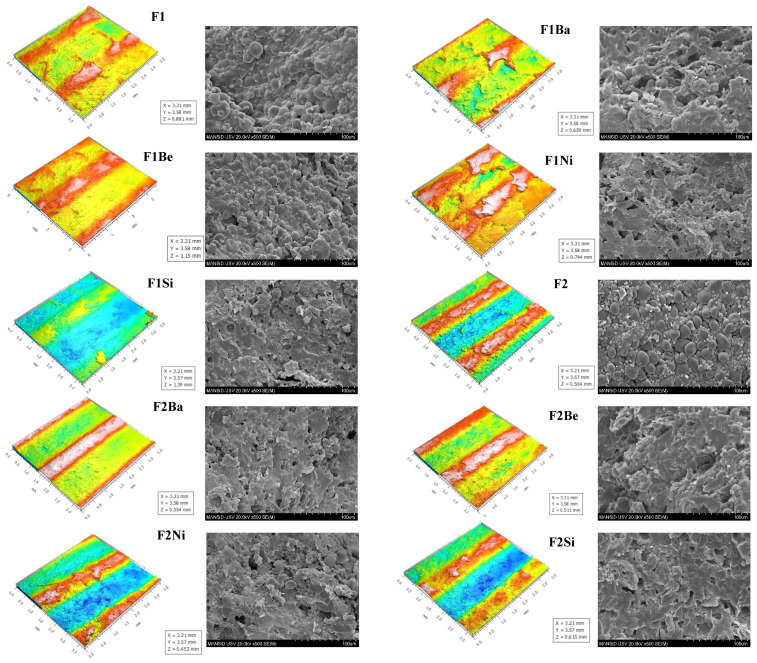
3D surface structure and SEM images of uncooked pasta with carrot pomace from different varieties.

The surface structure of pasta depends on a number of factors, such as the mold used, the moisture content of the dough, and the presence and size of solid particles. The 3D surface structure and microstructure of uncooked pasta with carrot pomace are shown in Figure 5. SEM images revealed the presence of carrot pomace particles in a compact structure of dough. Cooking of enriched pasta determined a continuous protein–starch matrix, with some interruptions caused by the presence of carrot pomace particles (Figure S11, Supplementary Materials). The SEM observations provide qualitative information regarding the structural organization of the samples and are intended to support the measured functional properties.

Compared with the control samples, the surface roughness (Figure S11, Supplementary Materials) decreased with the addition of carrot pomace, except for the F1Ni sample. The incorporation of pomace modifies the protein–starch matrix, causing the appearance of pores or discontinuities that can lead to a decrease in elasticity [58]. The fibers in carrot pomace with small particle sizes can fill the microspaces on the surface of the pasta, which leads to a more uniform and smoother texture, as supported by Bianchi et al. [42]. Carrot pomace contains bioactive components such as sugars and fibers that can influence the dough structure, making it more cohesive and less porous, which reduces the surface roughness of pasta after drying, especially since sugars can form bonds with proteins [93]. Ungureanu-Iuga and Mironeasa [94] reported an increase in the cohesiveness of pasta with the addition of grape skins.

The texture of cooked pasta obtained from the optimal mixtures of carrot pomace with wheat flours is presented in Table 5. Compared with the control samples, a decrease in the elasticity of the pasta was observed with the addition of carrot pomace, and the most pronounced changes were observed in the samples with higher doses (F2Ni, F2Si). An improvement in dough cohesion was observed with the incorporation of carrot pomace compared with the control samples. Also, compared with the control samples, significant increases in the gumminess, chewiness, and firmness of the pasta were observed depending on the dose of carrot pomace added. Carrot pomace is rich in fiber and can reduce gluten proteins (gliadin and glutenin), leading to the weakening of the gluten network that provides elasticity [77]. Also, the presence of polyphenols that interact with gluten proteins can affect the texture of pasta, as disulfide bonds can be disrupted and hydrophobic interactions of the dough can be modified [95]. In general, pasta with carrot pomace absorbed less water than the control sample, resulting in a denser internal structure and higher tensile strength, as also reported by Gałkowska et al. [77]. Previous studies have explained the increase in firmness by the presence of insoluble fibers, which can increase starch viscosity and slow down its retrogradation in the short term [45]. Chetrariu and Dabija [96] reported an increase in pasta firmness with the addition of malt by-product resulting from the manufacturing of spirits. The high fiber content of carrot pomace improves the water-holding capacity of the dough, which leads to better moisture retention [97] and, implicitly, to the better cohesiveness of the final product. In addition, the presence of carrot pomace delayed starch gelatinization during boiling (Figure 3), leading to a more gradual development of the pasta texture, with a firmer and more cohesive structure.

#### 3.3.4. Sensory Profile

**Figure 6:**
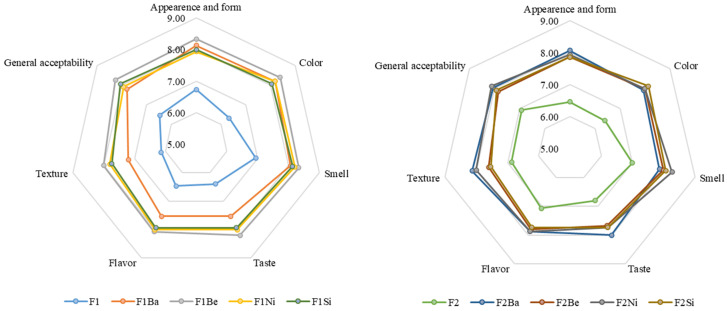
Sensory characteristics of the optimal pasta samples.

The sensory profile of pasta obtained from the optimal mixes of flour and carrot pomace is represented in Figure 6. An increase in the scores given to pasta with the addition of carrot pomace can be observed compared with the control samples, which obtained the lowest scores for all the studied characteristics. Sample F1Be (9.25%) from durum wheat flour and samples F2Ba (8.12%) and F2Ni (10.36%) respectively obtained the best scores for appearance and shape, color, odor, taste, aroma, texture, and overall acceptability. These results confirm the opportunity to use carrot pomace to obtain nutritionally improved pasta that is accepted by consumers. Another study confirmed that the addition of carrot puree to noodles led to improved scores for color, taste, and texture [98]. Fried instant noodles made from wheat flour with soybeans and carrot pomace showed superior sensory properties of color and taste compared with the control sample, according to data presented by Tiony and Irene [99]. Pasta made from flour with carrot pomace of various varieties can be considered acceptable (Figure 6) since the scores for overall acceptability were higher than 5 [53]. A study on the enrichment of common wheat pasta with carrot flour demonstrated that even at high doses (20%), the scores for sensory characteristics were comparable to those of the control sample, indicating that the inclusion of carrot flour did not negatively affect the perception or acceptance of the tasters [91]. Carrot pomace contains natural pigments, especially β-carotene [7], which give the pasta a pleasant orange hue and intensify the visual appearance, increasing the attractiveness of the product for consumers [91]. In addition, carrot flavor compounds can improve the taste and smell of the final product. The study by Rekha et al. [20] showed that the addition of carrot puree to common wheat flour improved the appearance, texture, aroma, and overall quality of the resulting pasta. The fiber from carrot pomace may contribute to a more consistent texture, providing a more satisfying chewing sensation, which is supported by the results obtained for the texture of the pasta (Table 5). The higher sensory scores for texture were consistent with the instrumental texture profile analysis results, whereas the color perception by panelists reflected the measured hue and yellowness values.

#### 3.3.5. Correlations and Principal Component Analysis

**Figure 7:**
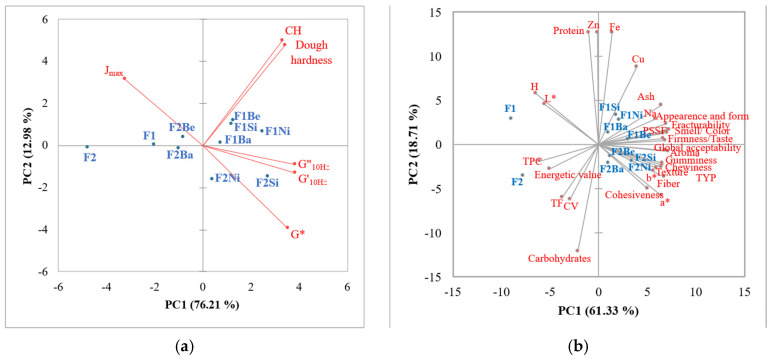
Graphical representation of the first two components according to the principal component analysis for the characteristics of dough (**a**) and pasta (**b**), with the addition of carrot pomace.

The principal component analysis applied to the characteristics of carrot pomace flour and pasta dough revealed that the first component (PC1) explains 76.21% of the data variation, and the second (PC2) explains 12.98% of the total variance (Figure 7a). The elastic moduli (G′) and viscosity (G″), as well as the maximum compliance (Jmax) are associated with PC1, while the HC, complex modulus (G*), and dough hardness are associated with PC2. A clustering of durum wheat flour dough samples is observed in the upper-right quadrant (Figure 7a). The control samples are located in the left quadrants and are differentiated mainly by the Jmax value. According to the principal component analysis applied to the characteristics of the finished product (Figure 7b), PC1 explained 61.33% of the data variation, and PC2 explained 18.71%. PC1 was associated with the textural properties, sensory properties, fiber content, Na, color parameters, total yellow pigment content, and TPC. PC2 was associated with the content of carbohydrates, proteins, Zn, Fe, Cu, OCT, and WA. The control samples are in opposition to each other and are located in the left quadrants compared with the samples with the addition of carrot pomace, which are located in the right quadrants. Common wheat flour samples with carrot pomace added are differentiated by appearance and shape, CL, fracturability, odor and color, firmness and taste, while common wheat flour samples with carrot pomace are distinguished by chewiness, gumminess, TYPs, a*, b*, and cohesiveness.

A series of correlations was observed between the dough characteristics, which are observed in Table S2 (Supplementary Materials). The values of the elastic and viscous moduli at a frequency of 10 Hz were positively correlated with the dough hardness and the flour HC (r > 0.65, *p* < 0.05) and negatively with Jmax (r > −0.66, *p* < 0.05). A significant positive correlation was observed between the dough hardness and HC (r = 0.88, *p* < 0.05) (Table S3, Supplementary Materials). Fe content was positively correlated with protein content (r = 0.91, *p* < 0.05). Cu content was positively correlated with the sensory parameters of smell and taste (r > 0.64, *p* < 0.05). Na content was positively correlated with CL, sensory parameters except texture, total yellow pigment content, fracturability, chewability, gumminess, firmness, and ash content (r > 0.64, *p* < 0.05), while with L* the correlation was negative (r > −0.64, *p* < 0.05). OCT was negatively correlated with firmness (r = −0.64, *p* < 0.05). Color parameters (a*, b*) were positively correlated with texture, sensory characteristics, fiber content and TYPs (r > 0.66, *p* < 0.05), while with TPC the correlation was negative (r > −0.64, *p* < 0.05). L* and H were negatively correlated with some sensory characteristics, especially those related to visual appearance (Table S3, Supplementary Materials), as well as with fiber content, firmness, and gumminess (r > −0.69, *p* < 0.05). Sensory properties were positively correlated with gumminess, firmness, chewiness, and fracturability of pasta (r > 0.63, *p* < 0.05). Fracturability was positively correlated with fiber content (r = 0.66, *p* < 0.05) and negatively with TPC (r = −0.70, *p* < 0.05). Fiber content was positively correlated with chewiness, gumminess, and firmness (r > 0.71, *p* < 0.05). Fiber content showed significant positive correlations with all texture parameters, arguing for the role of fibers in strengthening the structural matrix and achieving optimal consistency. Accordingly, sensory parameters were validated as predictors of texture through positive correlations with gumminess and firmness. These data support the hypothesis that mineral elements and fibers contribute to the modulation of organoleptic perception and structural integrity.

## 4. Conclusions

The incorporation of carrot pomace significantly affected dough rheology, pasta texture, cooking behavior, and nutritional composition, with the extent of changes depending on the carrot cultivar. Increasing pomace levels enhanced flour water absorption, dough viscoelasticity, crude fiber and pigment content, while reducing dough deformation resistance, pasta color intensity, and chewiness. Optimization identified cultivar-specific addition levels for durum and common wheat flours that ensured nutritional improvement without compromising quality. At these levels, all pasta samples showed acceptable cooking losses (<8%), intensified yellow coloration, and improved dietary fiber, particularly for Niagara and Sirkana cultivars. Overall, carrot pomace can be effectively used as a functional ingredient in pasta formulations, improving nutritional value and sensory acceptance while maintaining technological quality, thus supporting its application in innovative, value-added cereal products. Since the study was conducted using only two flour systems, the findings should be interpreted within the context of the evaluated materials. Further research involving additional wheat cultivars and quality classes is needed to confirm the broader applicability of the observed trends. A limitation of the present study is that only crude fiber content was determined. Future studies should investigate fiber fractions to better elucidate their specific contributions to dough rheology, water absorption, and pasta quality. In addition, further dynamic rheological measurements could be combined with alveograph analyses to obtain a more comprehensive understanding of the mechanisms underlying the effects of carrot pomace incorporation on dough structure, extensibility, and gluten functionality.

## Figures and Tables

**Table 1:** Influence of factors and fit of the mathematical model.

Factor	HC (%)	G* (Pa)	J_max_ (Pa·s)	Dough Hardness (N)	Chroma (adim.)	CL (%)	Fracturability (N)	Chewiness (N)	TYPs (mg/kg)	Fiber (%)
	*p*-Value									
Model	<0.01	<0.01	<0.01	<0.01	<0.01	<0.01	<0.01	<0.01	<0.01	<0.01
A	<0.01	<0.01	<0.01	<0.01	<0.01	<0.01	0.01	0.36	<0.01	0.01
B	<0.01	<0.01	<0.01	<0.01	<0.01	<0.01	<0.01	<0.01	<0.01	<0.01
C	<0.01	<0.01	<0.01	<0.01	<0.01	0.01	<0.01	<0.01	<0.01	<0.01
AB	<0.01	<0.01	<0.01	0.01	<0.01	0.61	0.12	0.93	<0.01	0.04
AC	<0.01	<0.01	<0.01	<0.01	<0.01	0.01	<0.01	<0.01	<0.01	0.11
BC	<0.01	<0.01	<0.01	0.97	<0.01	0.07	0.31	0.01	<0.01	0.01
*R* ^2^	0.98	0.84	0.84	0.89	0.91	0.84	0.85	0.75	0.90	0.88
Adj-*R*^2^	0.97	0.82	0.82	0.88	0.90	0.82	0.83	0.71	0.89	0.86
Pred.-*R*^2^	0.97	0.77	0.78	0.84	0.88	0.79	0.80	0.65	0.86	0.83
CV (%)	3.23	18.31	24.85	10.45	2.24	9.96	9.53	9.28	8.80	17.14

A—flour type-protein content, B—addition dose, C—carrot variety, HC—hydration capacity, G*—complex modulus, Jmax—maximum compliance, CL—solids loss upon boiling.

**Table 2:** Model validation.

Sample	Type of Values	F1Ba	F1Be	F1Ni	F1Si	F2Ba	F2Be	F2Ni	F2Si
Flour type-protein content (%)	-	F1	F1	F1	F1	F2	F2	F2	F2
Dose (%)	-	6.34	9.25	8.30	7.71	8.12	9.06	10.36	11.17
Carrot variety	-	Baltimore	Belgrado	Niagara	Sirkana	Baltimore	Belgrado	Niagara	Sirkana
HC (%)	predicted	75.30 ± 2.37 ^Ay^	83.67 ± 2.37 ^Bx^	87.02 ± 2.37 ^Ax^	88.25 ± 2.37 ^Ax^	75.94 ± 2.37 ^Ay^	65.86 ± 2.37 ^Azw^	63.07 ± 2.37 ^Aw^	71.52 ± 2.37 ^Ayz^
	experimental	74.87 ± 0.33 ^Ae^	88.25 ± 0.07 ^Aa^	83.18 ± 0.19 ^Ac^	81.00 ± 0.08 ^Ab^	69.54 ± 0.33 ^Be^	66.76 ± 0.30 ^Af^	65.18 ± 0.23 ^Ag^	74.76 ± 0.23 ^Ad^
G* (kPa)	predicted	591.69 ± 83.29 ^Axy^	437.86 ± 83.29 ^Ay^	546.33 ± 83.29 ^Axy^	461.80 ± 83.29 ^Ay^	514.98 ± 83.29 ^Axy^	422.05 ± 83.29 ^Ay^	505.28 ± 83.29 ^Axy^	714.86 ± 83.29 ^Ax^
	experimental	524.65 ± 2.75 ^Abcd^	469.80 ± 12.20 ^Acde^	535.60 ± 13.40 ^Abd^	456.80 ± 21.00 ^Ade^	511.02 ± 25.87 ^Abcd^	418.40 ± 4.00 ^Ae^	557.35 ± 13.95 ^Ab^	766.35 ± 60.35 ^Aa^
J_max_ (Pa·s)	predicted	3.80 ± 0.99 ^Axy^	2.25 ± 0.99 ^Ay^	2.59 ± 0.99 ^Ay^	3.33 ± 0.99 ^Axy^	3.92 ± 0.99 ^Axy^	5.39 ± 0.99 ^Ax^	2.33 ± 0.99 ^Ay^	1.73 ± 0.99 ^Ay^
	experimental	3.46 ± 0.29 ^Abc^	2.24 ± 0.94 ^Ade^	2.75 ± 0.05 ^Acd^	3.48 ± 0.24 ^Abd^	3.81 ± 0.38 ^Ab^	5.40 ± 0.11 ^Aa^	2.08 ± 0.25 ^Ade^	1.64 ± 0.49 ^Ae^
Dough hardness (N)	predicted	37.04 ± 3.40 ^Axyz^	39.67 ± 3.40 ^Axy^	44.49 ± 3.40 ^Ay^	35.76 ± 3.40 ^Axyz^	30.60 ± 3.40 ^Ayz^	28.28 ± 3.40 ^Az^	31.91 ± 3.40 ^Ayz^	32.74 ± 3.40 ^Ayz^
	experimental	39.00 ± 1.28 ^Aabc^	41.22 ± 4.39 ^Aab^	44.71 ± 1.81 ^Aa^	38.82 ± 4.64 ^Aabc^	27.49 ± 3.76 ^Ad^	29.52 ± 4.25 ^Acd^	25.35 ± 1.85 ^Bd^	33.20 ± 4.91 ^Abcd^
Chroma (adim.)	predicted	23.39 ± 0.50 ^Ax^	22.99 ± 0.50 ^Axy^	22.03 ± 0.50^Bxy^	23.26 ± 0.50 ^Axy^	20.13 ± 0.50^Bz^	19.95 ± 0.50 ^Az^	21.86 ± 0.50 ^By^	22.80 ± 0.50 ^Axy^
	experimental	21.30 ± 0.05 ^Bd^	20.03 ± 1.03 ^Bf^	24.91 ± 0.61 ^Aa^	22.72 ± 0.19 ^Abc^	22.15 ± 0.20 ^Ac^	20.36 ± 0.04 ^Ae^	23.21 ± 0.29 ^Ab^	22.28 ± 0.07 ^Ae^
CL (%)	predicted	6.82 ± 0.67 ^Axy^	7.58 ± 0.67 ^Axy^	7.65 ± 0.67 ^Axy^	7.71 ± 0.67 ^Axy^	6.10 ± 0.67 ^Ay^	6.38 ± 0.67 ^Axy^	8.12 ± 0.67 ^Ax^	7.02 ± 0.67 ^Axy^
	experimental	6.45 ± 0.23 ^Ab^	7.82 ± 0.36 ^Aa^	7.72 ± 0.64 ^Aa^	7.72 ± 0.33 ^Aa^	7.38 ± 0.00 ^Aa^	7.64 ± 0.34 ^Aa^	7.94 ± 0.03 ^Aa^	7.82 ± 0.00 ^Aa^
Fracturability (N)	predicted	52.28 ± 4.59 ^Axy^	60.66 ± 4.59 ^Ax^	50.75 ± 4.59 ^Axyz^	38.98 ± 4.59 ^Az^	44.77 ± 4.59 ^Ayz^	52.18 ± 4.59 ^Axy^	52.77 ± 4.59 ^Axy^	57.37 ± 4.59 ^Axy^
	experimental	58.54 ± 1.66 ^Aa^	60.48 ± 7.30 ^Aa^	49.98 ± 5.11 ^Aabc^	40.83 ± 6.05 ^Ac^	45.13 ± 0.76 ^Abc^	48.37 ± 2.68 ^Aabc^	54.82 ± 4.54 ^Aab^	58.48 ± 4.20 ^Aa^
Masticability (N)	predicted	24.15 ± 2.16 ^Axy^	24.52 ± 2.16 ^Axy^	20.81 ± 2.16 ^Axy^	21.79 ± 2.16 ^Axy^	19.62 ± 2.16 ^Ay^	19.00 ± 2.16 ^Ay^	24.88 ± 2.16 ^Axy^	26.59 ± 2.16 ^Ax^
	experimental	22.97 ± 1.09 ^Aab^	21.97 ± 1.65 ^Aab^	20.56 ± 0.28 ^Abc^	20.26 ± 0.28 ^Abc^	18.00 ± 0.54 ^Ac^	19.30 ± 1.04 ^Abc^	23.15 ± 0.91 ^Aab^	24.93 ± 2.95 ^Aa^
TYPs (mg/kg)	predicted	0.30 ± 0.03 ^Ay^	0.29 ± 0.03 ^Ay^	0.36 ± 0.03 ^Ay^	0.35 ± 0.03 ^Ay^	0.29 ± 0.03 ^Ay^	0.33 ± 0.03 ^Ay^	0.53 ± 0.03 ^Ax^	0.49 ± 0.03 ^Ax^
	experimental	0.27 ± 0.01 ^Ae^	0.34 ± 0.00 ^Ac^	0.36 ± 0.01 ^Ab^	0.35 ± 0.00 ^Ac^	0.30 ± 0.00 ^Ad^	0.34 ± 0.00 ^Ac^	0.51 ± 0.00 ^Aa^	0.50 ± 0.00 ^Aa^
Crude fiber (%)	predicted	0.77 ± 0.17 ^Az^	0.89 ± 0.17 ^Ayz^	1.03 ± 0.17 ^Ayz^	1.20 ± 0.17 ^Axyz^	1.05 ± 0.17 ^Ayz^	1.10 ± 0.17 ^Ayz^	1.29 ± 0.17 ^Axy^	1.66 ± 0.17 ^Ax^
	experimental	0.81 ± 0.01 ^Ad^	0.93 ± 0.02 ^Ad^	1.09 ± 0.02 ^Ac^	1.18 ± 0.03 ^Ac^	1.11 ± 0.03 ^Ac^	1.17 ± 0.02 ^Ac^	1.40 ± 0.08 ^Ab^	1.65 ± 0.10 ^Aa^

F1—durum wheat flour (14% protein), F2—common wheat flour (11% protein), A,B—distinct letters in the same column for each characteristic suggest significant differences between predicted and experimental values (*p* < 0.05), a–g—lowercase letters in the same row suggest significant differences (*p* < 0.05) between samples (experimental values), w–z—lowercase letters in the same row suggest significant differences (*p* < 0.05) between samples (predicted values); Ba—Baltimore, Be—Belgrado, Ni—Niagara, Si—Sirkana, HC—hydration capacity, G*—complex modulus, Jmax—maximum creep compliance, CL—solids loss on boiling, TYPs—total yellow pigments.

**Table 3:** Chemical composition of cooked pasta obtained from optimal mixes.

Sample	Protein (% d.w.)	Lipid (% d.w.)	Ash (% d.w.)	Crude Fiber (% d.w.)	Carbohydrates (% d.w.)	Energetic Value (kcal/100 g)	TPC (mg GAE/100 g d.w.)
F1	14.57 ± 0.03 ^b^	0.78 ± 0.04 ^d^	0.95 ± 0.03 ^f^	0.64 ± 0.03 ^e^	83.05 ± 0.14 ^d^	357.26 ± 0.04 ^b^	15.25 ± 0.09 ^a^
F1Ba	14.02 ± 0.03 ^c^	0.75 ± 0.03 ^d^	1.32 ± 0.02 ^c^	0.90 ± 0.01 ^d^	83.01 ± 0.03 ^d^	355.74 ± 0.13 ^c^	11.16 ± 0.04 ^f^
F1Be	12.72 ± 0.02 ^d^	0.88 ± 0.02 ^c^	1.60 ± 0.02 ^a^	1.04 ± 0.02 ^d^	83.76 ± 0.04 ^c^	354.33 ± 0.05 ^d^	10.72 ± 0.23 ^g^
F1Ni	14.52 ± 0.03 ^b^	0.74 ± 0.01 ^d^	1.43 ± 0.02 ^b^	1.22 ± 0.02 ^c^	82.08 ± 0.08 ^e^	353.21 ± 0.16 ^f^	12.39 ± 0.07 ^d^
F1Si	14.69 ± 0.02 ^a^	0.72 ± 0.04 ^d^	1.48 ± 0.01 ^b^	1.31 ± 0.03 ^c^	81.79 ± 0.05 ^f^	353.36 ± 0.17 ^ef^	10.28 ± 0.08 ^h^
F2	12.13 ± 0.02 ^g^	1.19 ± 0.02 ^b^	0.54 ± 0.02 ^g^	0.63 ± 0.02 ^e^	85.51 ± 0.00 ^a^	358.94 ± 0.02 ^a^	14.80 ± 0.04 ^b^
F2Ba	12.43 ± 0.03 ^e^	0.50 ± 0.03 ^e^	1.11 ± 0.02 ^e^	1.22 ± 0.03 ^c^	84.73 ± 0.10 ^b^	354.00 ± 0.01 ^d^	11.11 ± 0.04 ^f^
F2Be	12.24 ± 0.03 ^f^	1.40 ± 0.04 ^a^	1.25 ± 0.02 ^d^	1.30 ± 0.02 ^c^	83.81 ± 0.02 ^c^	358.93 ± 0.35 ^a^	11.96 ± 0.10 ^e^
F2Ni	12.39 ± 0.03 ^e^	0.52 ± 0.03 ^e^	1.61 ± 0.01 ^a^	1.56 ± 0.09 ^b^	83.91 ± 0.02 ^c^	351.61 ± 0.20 ^g^	10.97 ± 0.08 ^fg^
F2Si	12.40 ± 0.03 ^e^	0.51 ± 0.04 ^e^	1.44 ± 0.01 ^b^	1.83 ± 0.11 ^a^	83.81 ± 0.03 ^c^	353.88 ± 0.32 ^de^	13.24 ± 0.09 ^c^

F1—durum wheat flour, F2—common wheat flour, F1Ba—6.34% Baltimore, F1Be—9.25% Belgrado, F1Ni—8.30% Niagara, F1Si—7.71% Sirkana, F2Ba—8.12% Baltimore, F2Be—9.06% Belgrado, F2Ni—10.36% Niagara, F2Si—11.17% Sirkana, TPC—total polyphenol content, d.w.—dry matter; a–g—distinct letters in the same column indicate significant differences between samples (*p* < 0.05).

**Table 4:** Color and cooking behavior.

Sample	Cooking Behavior			Uncooked Pasta Color				Cooked Pasta Color			
	OCT (min)	CL (%)	WA (%)	L* (adim.)	a* (adim.)	b* (adim.)	H (adim.)	L* (adim.)	a* (adim.)	b* (adim.)	H (adim.)
F1	12.50 ± 0.00 ^b^	5.00 ± 0.30 ^c^	130.77 ± 7.71 ^c^	71.71 ± 0.39 ^b^	0.06 ± 0.05 ^d^	17.64 ± 0.17 ^f^	89.82 ± 0.14 ^a^	78.06 ± 1.27 ^a^	−1.35 ± 0.28 ^h^	14.59 ± 0.32 ^h^	95.28 ± 1.00 ^a^
F1Ba	12.25 ± 0.25 ^bc^	6.45 ± 0.23 ^b^	133.36 ± 9.53 ^c^	75.83 ± 0.72 ^a^	0.41 ± 0.09 ^cd^	21.30 ± 0.05 ^cd^	88.89 ± 0.24 ^ab^	66.64 ± 0.44 ^e^	1.70 ± 0.21 ^ef^	21.87 ± 0.28 ^f^	85.57 ± 0.49 ^c^
F1Be	12.00 ± 0.00 ^c^	7.82 ± 0.36 ^a^	128.27 ± 1.30 ^c^	66.30 ± 0.84 ^cd^	0.66 ± 0.24 ^c^	20.02 ± 1.03 ^e^	88.13 ± 0.65 ^bc^	72.65 ± 0.24 ^c^	2.84 ± 0.08 ^d^	26.67 ± 0.23 ^cd^	83.93 ± 0.13 ^d^
F1Ni	12.50 ± 0.00 ^b^	7.72 ± 0.64 ^a^	133.41 ± 0.25 ^c^	71.15 ± 2.78 ^b^	1.88 ± 0.24 ^a^	24.83 ± 0.63 ^a^	85.67 ± 0.65 ^de^	72.38 ± 0.98 ^c^	1.99 ± 0.20 ^e^	25.63 ± 0.05 ^e^	85.57 ± 0.44 ^c^
F1Si	12.00 ± 0.00 ^c^	7.72 ± 0.33 ^a^	133.28 ± 3.21 ^c^	68.98 ± 0.56 ^bc^	1.91 ± 0.02 ^a^	22.64 ± 0.19 ^b^	85.19 ± 0.05 ^e^	72.40 ± 0.15 ^c^	1.52 ± 0.11 ^f^	26.53 ± 0.44 ^cd^	86.72 ± 0.17 ^c^
F2	13.25 ± 0.25 ^a^	4.36 ± 0.06 ^c^	168.54 ± 8.43 ^a^	65.80 ± 0.31 ^d^	0.52 ± 0.10 ^cd^	16.07 ± 0.04 ^g^	88.16 ± 0.34 ^b^	75.52 ± 0.20 ^b^	0.18 ± 0.01 ^g^	19.95 ± 0.18 ^g^	89.49 ± 0.02 ^b^
F2Ba	13.25 ± 0.25 ^a^	7.38 ± 0.00 ^a^	125.15 ± 2.42 ^c^	63.98 ± 0.65 ^d^	2.10 ± 0.25 ^a^	22.05 ± 0.18 ^bc^	84.57 ± 0.60 ^e^	68.68 ± 0.18 ^d^	3.81 ± 0.06 ^ab^	27.46 ± 0.04 ^b^	82.11 ± 0.12 ^e^
F2Be	12.00 ± 0.00 ^c^	7.64 ± 0.34 ^a^	123.87 ± 1.28 ^c^	64.55 ± 0.42 ^d^	1.90 ± 0.05 ^a^	20.27 ± 0.04 ^de^	84.66 ± 0.12 ^e^	67.32 ± 0.47 ^de^	3.27 ± 0.09 ^cd^	26.11 ± 0.43 ^d^	82.87 ± 0.08 ^de^
F2Ni	12.50 ± 0.00 ^b^	7.94 ± 0.03 ^a^	135.01 ± 6.78 ^c^	63.58 ± 0.27 ^d^	1.28 ± 0.33 ^b^	23.17 ± 0.28 ^b^	86.85 ± 0.78 ^cd^	68.38 ± 0.22 ^d^	3.36 ± 0.22 ^bc^	27.18 ± 0.16 ^bc^	82.96 ± 0.41 ^de^
F2Si	12.00 ± 0.00 ^c^	7.82 ± 0.00 ^a^	150.83 ± 0.55 ^b^	63.96 ± 0.54 ^d^	1.22 ± 0.01 ^b^	22.24 ± 0.07 ^bc^	86.85 ± 0.01 ^cd^	67.81 ± 0.17 ^de^	3.95 ± 0.11 ^a^	29.01 ± 0.10 ^a^	82.25 ± 0.19 ^e^

F1—durum wheat flour, F2—common wheat flour, F1Ba—6.34% Baltimore, F1Be—9.25% Belgrado, F1Ni—8.30% Niagara, F1Si—7.71% Sirkana, F2Ba—8.12% Baltimore, F2Be—9.06% Belgrado, F2Ni—10.36% Niagara, F2Si—11.17% Sirkana, OCT—optimal boiling time, CL—solids loss during cooking, WA—water absorption; a–h—distinct letters in the same column indicate significant differences between samples (*p* < 0.05).

**Table 5:** Cooked pasta texture parameters.

Sample	Chewiness (N)	Elasticity (%)	Cohesiveness (adim.)	Gumminess (N)	Firmness (N)
F1	10.51 ± 0.15 ^f^	99.98 ± 0.21 ^a^	0.36 ± 0.02 ^c^	10.53 ± 0.14 ^f^	27.64 ± 2.35 ^bc^
F1Ba	22.97 ± 1.09 ^abc^	99.95 ± 0.09 ^a^	0.67 ± 0.07 ^ab^	23.03 ± 1.08 ^ab^	32.90 ± 2.58 ^a^
F1Be	21.97 ± 1.65 ^abc^	99.84 ± 0.01 ^ab^	0.67 ± 0.01 ^ab^	21.45 ± 1.50 ^abc^	32.01 ± 0.53 ^ab^
F1Ni	20.56 ± 0.28 ^bcd^	99.84 ± 0.00 ^ab^	0.60 ± 0.03 ^b^	20.62 ± 0.28 ^bcd^	33.30 ± 0.90 ^a^
F1Si	20.26 ± 0.28 ^bcd^	99.83 ± 0.01 ^ab^	0.66 ± 0.04 ^ab^	20.34 ± 0.27 ^bcd^	31.70 ± 2.07 ^ab^
F2	16.20 ± 1.10 ^e^	99.96 ± 0.07 ^a^	0.65 ± 0.03 ^ab^	16.23 ± 1.10 ^e^	25.48 ± 1.97 ^c^
F2Ba	18.00 ± 0.54 ^de^	99.84 ± 0.23 ^ab^	0.59 ± 0.04 ^b^	18.04 ± 0.55 ^de^	30.72 ± 1.37 ^ab^
F2Be	19.30 ± 1.04 ^cde^	99.65 ± 0.01 ^b^	0.61 ± 0.01 ^b^	19.38 ± 1.02 ^cde^	33.86 ± 2.00 ^a^
F2Ni	23.15 ± 0.91 ^ab^	99.68 ± 0.00 ^ab^	0.63 ± 0.04 ^ab^	22.68 ± 0.00 ^ab^	34.57 ± 0.00 ^a^
F2Si	24.93 ± 2.95 ^a^	99.83 ± 0.00 ^ab^	0.74 ± 0.06 ^a^	23.99 ± 2.49 ^a^	35.30 ± 0.43 ^a^

F1—durum wheat flour, F2—common wheat flour, F1Ba—6.34% Baltimore, F1Be—9.25% Belgrado, F1Ni—8.30% Niagara, F1Si—7.71% Sirkana, F2Ba—8.12% Baltimore, F2Be—9.06% Belgrado, F2Ni—10.36% Niagara, F2Si—11.17% Sirkana; a–f—distinct letters in the same column indicate significant differences between samples (*p* < 0.05).

## Data Availability

The raw data supporting the conclusions of this article will be made available by the authors on request.

## Supplementary Materials

The following supporting information can be downloaded at: https://www.mdpi.com/article/10.3390/foods15122201/s1, Figure S1. Combined effect of flour type (protein content) and carrot pomace dose from different varieties: (a) Baltimore, (b) Belgrado, (c) Niagara, (d) Sirkana on flour hydration capacity; Figure S2. The combined effect of flour type (protein content) and carrot pomace dosage from different varieties: (a) Baltimore, (b) Belgrado, (c) Niagara, (d) Sirkana on the complex modulus of dough (G*); Figure S3. Combined effect of flour type (protein content) and carrot pomace dosage of different varieties: (a) Baltimore, (b) Belgrado, (c) Niagara, (d) Sirkana on maximum dough compliance (Jmax); Figure S4. The combined effect of flour type (protein content) and carrot pomace dosage from different varieties: (a) Baltimore, (b) Belgrado, (c) Niagara, (d) Sirkana on dough hardness; Figure S5. The combined effect of flour type (protein content) and carrot pomace dosage from different varieties: (a) Baltimore, (b) Belgrado, (c) Niagara, (d) Sirkana on the color (Chroma) of pasta; Figure S6. The combined effect of flour type (protein content) and carrot pomace dosage from different varieties: (a) Baltimore, (b) Belgrado, (c) Niagara, (d) Sirkana on the loss of soluble solids (CL) of pasta; Figure S7. The combined effect of flour type (protein content) and carrot pomace dosage from different varieties: (a) Baltimore, (b) Belgrado, (c) Niagara, (d) Sirkana on the fracturability of pasta; Figure S8. The combined effect of flour type (protein content) and carrot pomace dosage from different varieties: (a) Baltimore, (b) Belgrado, (c) Niagara, (d) Sirkana on the chewiness of pasta; Figure S9. The combined effect of flour type (protein content) and carrot pomace dosage from different varieties: (a) Baltimore, (b) Belgrado, (c) Niagara, (d) Sirkana on the total yellow pigment content of pasta; Figure S10. The combined effect of flour type (protein content) and carrot pomace dosage from different varieties: (a) Baltimore, (b) Belgrado, (c) Niagara, (d) Sirkana on the crude fiber content of pasta; Figure S11. The microstructure of cooked pasta and surface roughness (Rz) of uncooked pasta; Table S1. Experimental data used for mathematical modeling; Table S2. Correlations between flour and dough characteristics; Table S3. Correlations between the characteristics of the final product.
