# Supplementary material for: Rheological, Microstructural, and Physicochemical Characterization of Pasta Fortified with Carrot Pomace: A Comparative Study of Wheat Types and Carrot Varieties
Source: Foods. 2026 Jun 18;15(12):2201. doi: 10.3390/foods15122201 (PMC13298148; doi:10.3390/foods15122201)
Supplement: Supplementary file 1 [file foods-15-02201-s001.zip › foods-4343887-supplementary.pdf]

# SUPPLEMENTARY MATERIAL

**Table S1.** Experimental data used for mathematical modelling

| Sample | HC (%) | G* (Pa) | $J_{max}$ (Pa·s) | Dough hardness (N) | Chroma | CL (%) | Fracturability (N) | Chewiness (N) | TYP (mg/kg) | Fiber (%) |
|---|---|---|---|---|---|---|---|---|---|---|
| F1Ba3 | 62.42±0.16$^{aAz}$ | 376400±33876$^{aAz}$ | 4.30±0.20$^{bAx}$ | 31.65±3.74$^{aAz}$ | 25.16±0.03$^{aBx}$ | 4.97±0.20$^{aAz}$ | 38.96±3.58$^{aAw}$ | 26.37±2.15$^{aBx}$ | 0.28±0.00$^{aBz}$ | 0.51±0.07$^{aBw}$ |
| F1Ba6 | 79.80±0.14$^{aAy}$ | 591600±53244$^{aAz}$ | 3.70±0.20$^{bAy}$ | 33.12±1.72$^{aAyz}$ | 24.30±0.06$^{aBxy}$ | 7.15±0.55$^{aAy}$ | 50.83±4.27$^{aAz}$ | 24.70±2.36$^{aBx}$ | 0.32±0.00$^{aByz}$ | 0.53±0.06$^{aBz}$ |
| F1Ba9 | 84.24±0.10$^{aAxy}$ | 797200±71748$^{aAy}$ | 3.38±0.13$^{bAyz}$ | 40.75±0.98$^{aAy}$ | 21.35±0.26$^{aByz}$ | 7.87±0.08$^{aAy}$ | 64.15±3.79$^{aAy}$ | 23.56±1.91$^{aBxy}$ | 0.30±0.00$^{aBy}$ | 0.83±0.04$^{aBy}$ |
| F1Ba12 | 90.82±0.14$^{aAx}$ | 827300±74457$^{aAx}$ | 3.27±0.11$^{bAz}$ | 49.82±3.95$^{aAx}$ | 20.86±0.02$^{aBz}$ | 8.94±0.06$^{aAx}$ | 69.00±5.46$^{aAx}$ | 20.20±1.32$^{aBy}$ | 0.32±0.00$^{aBx}$ | 1.73±0.14$^{aBx}$ |
| F1Be3 | 69.96±0.11$^{aAz}$ | 302500±27225$^{aBz}$ | 3.79±0.10$^{bAx}$ | 31.46±2.79$^{aAz}$ | 24.47±0.20$^{aBx}$ | 5.70±0.35$^{aAz}$ | 44.42±3.38$^{aAw}$ | 28.97±2.17$^{aABx}$ | 0.25±0.00$^{aBz}$ | 0.26±0.04$^{aBw}$ |
| F1Be6 | 75.67±0.13$^{aAy}$ | 398300±35847$^{aBz}$ | 3.32±0.14$^{bAy}$ | 33.83±3.42$^{aAyz}$ | 24.38±0.41$^{aBxy}$ | 6.00±0.05$^{aAy}$ | 47.78±4.16$^{aAz}$ | 28.85±1.98$^{aABx}$ | 0.28±0.00$^{aByz}$ | 0.49±0.06$^{aBz}$ |
| F1Be9 | 82.42±0.15$^{aAxy}$ | 416800±37512$^{aBy}$ | 2.59±0.10$^{bAyz}$ | 37.44±0.42$^{aAy}$ | 23.74±0.03$^{aByz}$ | 6.78±0.33$^{aAy}$ | 60.83±5.06$^{aAy}$ | 26.07±2.44$^{aABxy}$ | 0.30±0.01$^{aBy}$ | 0.53±0.06$^{aBy}$ |
| F1Be12 | 90.03±0.09$^{aAx}$ | 535000±48150$^{aBx}$ | 2.19±0.18$^{bAz}$ | 45.73±1.91$^{aAx}$ | 21.57±0.01$^{aBz}$ | 8.98±0.07$^{aAx}$ | 69.01±4.94$^{aAx}$ | 20.02±2.15$^{aABy}$ | 0.31±0.01$^{aBx}$ | 1.50±0.09$^{aBx}$ |
| F1Ni3 | 70.64±0.24$^{aAz}$ | 464500±41805$^{aABz}$ | 3.43±0.14$^{bAx}$ | 39.98±0.84$^{aAz}$ | 23.67±0.40$^{aABx}$ | 5.34±0.11$^{aAz}$ | 38.25±2.93$^{aAw}$ | 22.25±1.77$^{aABx}$ | 0.28±0.01$^{aAz}$ | 0.48±0.06$^{aBw}$ |
| F1Ni6 | 77.77±0.11$^{aAy}$ | 519000±46710$^{aABz}$ | 3.29±0.21$^{bAy}$ | 41.91±1.11$^{aAyz}$ | 22.22±0.01$^{aABxy}$ | 6.51±0.58$^{aAy}$ | 49.04±3.97$^{aAz}$ | 19.95±1.86$^{aABx}$ | 0.32±0.00$^{aAyz}$ | 0.64±0.06$^{aBz}$ |
| F1Ni9 | 92.20±0.09$^{aAxy}$ | 531100±47799$^{aABy}$ | 2.04±0.03$^{bAyz}$ | 45.88±4.17$^{aAy}$ | 21.59±0.13$^{aAByz}$ | 8.21±0.26$^{aAy}$ | 51.84±3.17$^{aAy}$ | 19.46±0.87$^{aABxy}$ | 0.38±0.00$^{aAy}$ | 1.13±0.06$^{aBy}$ |
| F1Ni12 | 97.89±0.10$^{aAx}$ | 576200±51858$^{aABx}$ | 1.78±0.05$^{bAz}$ | 45.26±3.74$^{aAx}$ | 21.46±0.09$^{aABz}$ | 9.06±0.31$^{aAx}$ | 56.34±5.17$^{aAx}$ | 22.11±2.09$^{aABy}$ | 0.39±0.01$^{aAx}$ | 1.51±0.08$^{aBx}$ |
| F1Si3 | 67.38±0.19$^{aAz}$ | 249400±22446$^{aABz}$ | 5.36±0.24$^{bAx}$ | 27.78±1.79$^{aAz}$ | 24.06±0.30$^{aAx}$ | 6.03±0.90$^{aAz}$ | 26.09±2.10$^{aAw}$ | 23.33±1.94$^{aAx}$ | 0.32±0.01$^{aAz}$ | 1.08±0.01$^{aAw}$ |
| F1Si6 | 82.19±0.13$^{aAy}$ | 274000±24660$^{aABz}$ | 3.75±0.09$^{bAy}$ | 32.25±0.85$^{aAyz}$ | 23.54±0.48$^{aAxy}$ | 7.30±0.87$^{aAy}$ | 34.75±2.33$^{aAz}$ | 22.04±2.30$^{aAx}$ | 0.33±0.01$^{aAyz}$ | 1.16±0.05$^{aAz}$ |
| F1Si9 | 98.45±0.21$^{aAxy}$ | 569100±51219$^{aABy}$ | 2.43±0.19$^{bAyz}$ | 39.64±1.06$^{aAy}$ | 22.87±0.21$^{aAyz}$ | 8.21±0.81$^{aAy}$ | 38.39±2.90$^{aAy}$ | 21.83±0.38$^{aAxy}$ | 0.37±0.01$^{aAy}$ | 1.17±0.03$^{aAy}$ |
| F1Si12 | 101.94±0.16$^{aAx}$ | 711400±64026$^{aABx}$ | 2.03±0.10$^{bAz}$ | 42.15±2.87$^{aAx}$ | 22.77±0.11$^{aAz}$ | 9.03±0.83$^{aAx}$ | 54.22±4.27$^{aAx}$ | 20.45±0.99$^{aAy}$ | 0.39±0.01$^{aAx}$ | 1.36±0.03$^{aAx}$ |

*Cont.*

| Sample | HC (%) | G* (Pa) | $J_{max}$ (Pa·s) | Dough hardness (N) | Chroma | CL (%) | Fracturability (N) | Chewiness (N) | TYP (mg/kg) | Fiber (%) |
|---|---|---|---|---|---|---|---|---|---|---|
| F2Ba3 | 59.92±0.11$^{bAz}$ | 241600±21744$^{bAz}$ | 8.05±0.70$^{aAx}$ | 20.53±0.82$^{bAz}$ | 20.73±0.46$^{bBx}$ | 4.74±0.18$^{bAz}$ | 32.77±1.64$^{aAw}$ | 20.99±1.30$^{aBx}$ | 0.24±0.00$^{aBz}$ | 0.45±0.02$^{aBw}$ |
| F2Ba6 | 71.43±0.13$^{bAy}$ | 320600±28854$^{bAz}$ | 3.58±0.20$^{aAy}$ | 28.56±1.76$^{bAyz}$ | 20.39±0.48$^{bBxy}$ | 5.45±0.48$^{bAy}$ | 41.42±3.97$^{aAz}$ | 19.30±1.64$^{aBx}$ | 0.26±0.01$^{aByz}$ | 0.87±0.01$^{aBz}$ |
| F2Ba9 | 80.32±0.11$^{bAxy}$ | 571300±51417$^{bAy}$ | 2.87±0.16$^{aAyz}$ | 33.28±2.81$^{bAy}$ | 20.18±0.42$^{bByz}$ | 6.03±0.67$^{bAy}$ | 46.58±4.48$^{aAy}$ | 18.92±1.48$^{aBxy}$ | 0.29±0.00$^{aBy}$ | 1.17±0.03$^{aBy}$ |
| F2Ba12 | 86.09±0.07$^{bAx}$ | 767400±69066$^{bAx}$ | 2.60±0.03$^{aAz}$ | 34.81±0.94$^{bAx}$ | 19.78±0.06$^{bBz}$ | 7.33±0.42$^{bAx}$ | 51.96±4.07$^{aAx}$ | 20.20±2.00$^{aBy}$ | 0.35±0.00$^{aBx}$ | 1.42±0.03$^{aBx}$ |
| F2Be3 | 56.23±0.09$^{bAz}$ | 315000±28350$^{bBz}$ | 12.64±0.90$^{aAx}$ | 15.22±1.35$^{bAz}$ | 20.68±0.25$^{bBx}$ | 2.93±0.01$^{bAz}$ | 35.02±3.37$^{aAw}$ | 22.39±1.31$^{aABx}$ | 0.24±0.00$^{aBz}$ | 0.39±0.02$^{aBw}$ |
| F2Be6 | 62.66±0.13$^{bAy}$ | 336700±30303$^{bBz}$ | 7.46±0.52$^{aAy}$ | 23.44±0.64$^{bAyz}$ | 20.40±0.63$^{bBxy}$ | 6.35±0.78$^{bA}$y | 47.03±2.82$^{aAz}$ | 21.64±1.09$^{aABx}$ | 0.29±0.00$^{aByz}$ | 0.64±0.03$^{aBz}$ |
| F2Be9 | 66.39±0.09$^{bAxy}$ | 343100±30879$^{bBy}$ | 3.98±0.18$^{aAyz}$ | 29.40±2.44$^{bAy}$ | 19.85±0.11$^{bByz}$ | 6.42±0.52$^{bAy}$ | 52.06±4.24$^{aAy}$ | 21.23±0.48$^{aABxy}$ | 0.33±0.01$^{aBy}$ | 1.04±0.03$^{aBy}$ |
| F2Be12 | 69.85±0.06$^{bAx}$ | 509800±45882$^{bBx}$ | 2.88±0.27$^{aAz}$ | 32.48±2.21$^{bAx}$ | 19.64±0.19$^{bBz}$ | 7.38±0.27$^{bAx}$ | 58.96±5.66$^{aAx}$ | 15.86±0.22$^{aABy}$ | 0.36±0.01$^{aBx}$ | 1.50±0.06$^{aBx}$ |
| F2Ni3 | 49.17±0.07$^{bAz}$ | 198700±17883$^{bABz}$ | 4.67±0.32$^{aAx}$ | 14.91±0.74$^{bAz}$ | 22.13±0.08$^{bABx}$ | 4.19±0.09$^{bAz}$ | 32.20±2.82$^{aAw}$ | 26.25±4.80$^{aABx}$ | 0.28±0.01$^{aAz}$ | 0.36±0.03$^{aBw}$ |
| F2Ni6 | 56.37±0.09$^{bAy}$ | 216200±19458$^{bABz}$ | 5.82±0.29$^{aAy}$ | 21.60±0.49$^{bAyz}$ | 22.48±0.03$^{bABxy}$ | 7.21±0.51$^{bAy}$ | 50.40±4.38$^{aAz}$ | 26.14±1.37$^{aABx}$ | 0.34±0.01$^{aAyz}$ | 0.67±0.06$^{aBz}$ |
| F2Ni9 | 59.15±0.08$^{bAxy}$ | 301500±27135$^{bABy}$ | 2.80±0.10$^{aAyz}$ | 23.49±0.98$^{bAy}$ | 21.97±0.03$^{bAByz}$ | 7.42±0.15$^{bAy}$ | 51.78±4.30$^{aAy}$ | 24.96±1.90$^{aABxy}$ | 0.43±0.00$^{aAy}$ | 1.13±0.02$^{aBy}$ |
| F2Ni12 | 65.43±0.10$^{bAx}$ | 794800±71534$^{bABx}$ | 1.94±0.14$^{aAz}$ | 43.83±2.40$^{bAx}$ | 21.55±0.38$^{bABz}$ | 8.72±1.28$^{bAx}$ | 55.04±5.04$^{aAx}$ | 23.86±1.59$^{aABy}$ | 0.66±0.01$^{aAx}$ | 1.48±0.05$^{aBx}$ |
| F2Si3 | 52.87±0.07$^{bAz}$ | 230400±20736$^{bABz}$ | 8.55±0.67$^{aAx}$ | 18.57±0.66$^{bAz}$ | 23.69±0.24$^{bAx}$ | 5.01±0.08$^{bAz}$ | 33.84±2.61$^{aAw}$ | 33.31±3.07$^{aAx}$ | 0.28±0.01$^{aAz}$ | 0.63±0.02$^{aAw}$ |
| F2Si6 | 59.13±0.20$^{bAy}$ | 358500±32265$^{bABz}$ | 3.52±0.39$^{aAy}$ | 22.37±0.36$^{bAyz}$ | 23.45±0.40$^{bAxy}$ | 5.51±0.24$^{bAy}$ | 50.01±4.38$^{aAz}$ | 28.99±2.70$^{aAx}$ | 0.28±0.01$^{aAyz}$ | 1.35±0.04$^{aAz}$ |
| F2Si9 | 64.29±0.12$^{bAxy}$ | 498900±44901$^{bABy}$ | 3.88±0.13$^{aAyz}$ | 26.63±1.08$^{bAy}$ | 22.61±0.22$^{bAyz}$ | 5.72±0.25$^{bAy}$ | 51.73±3.41$^{aAy}$ | 27.66±1.14$^{aAxy}$ | 0.41±0.00$^{aAy}$ | 1.47±0.02$^{aAy}$ |
| F2Si12 | 72.46±0.06$^{bAx}$ | 797600±71784$^{bABx}$ | 1.87±0.02$^{aAz}$ | 34.33±1.90$^{bAx}$ | 22.34±0.30$^{bAz}$ | 7.60±0.04$^{bAx}$ | 57.68±5.25$^{aAx}$ | 24.94±2.50$^{aAy}$ | 0.56±0.00$^{aAx}$ | 1.88±0.06$^{aAx}$ |

F1 - durum wheat flour with 14% protein content, F2 - common wheat flour with 11% protein content, Ba - Baltimore, Be - Belgrado, Ni - Niagara, Si - Sirkana, HC - hydration capacity, G* - complex modulus, Jmax - maximum compliance, CL - loss of solids on boiling, TYP – total yellow pigments, 3/6/9/12% - addition levels of carrot pomace; a-b - average values followed by lowercase letters in the same column indicate significant differences between flour types ($p < 0.05$), A-B - average values followed by uppercase letters in the same column indicate significant differences between carrot varieties ($p < 0.05$), x-w - average values followed by lowercase letters in the same column indicate significant differences between doses ($p < 0.05$)

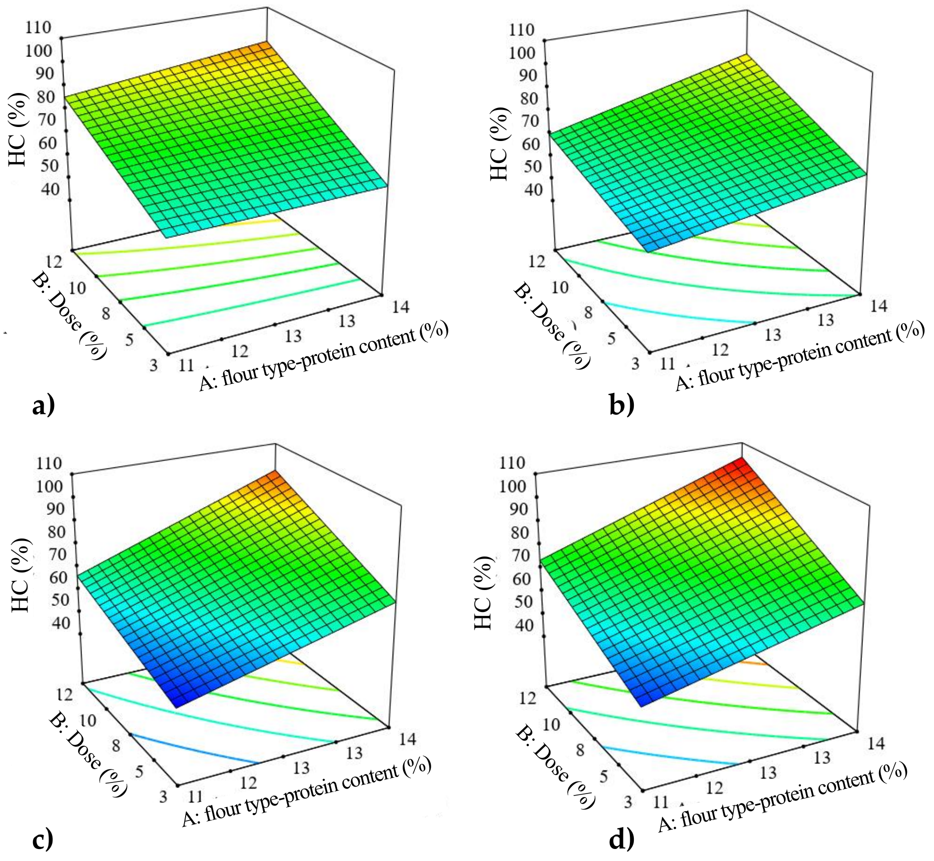


**Figure S.1.** Combined effect of flour type (protein content) and carrot pomace dose from different varieties: a) Baltimore, b) Belgrado, c) Niagara, d) Sirkana on flour hydration capacity

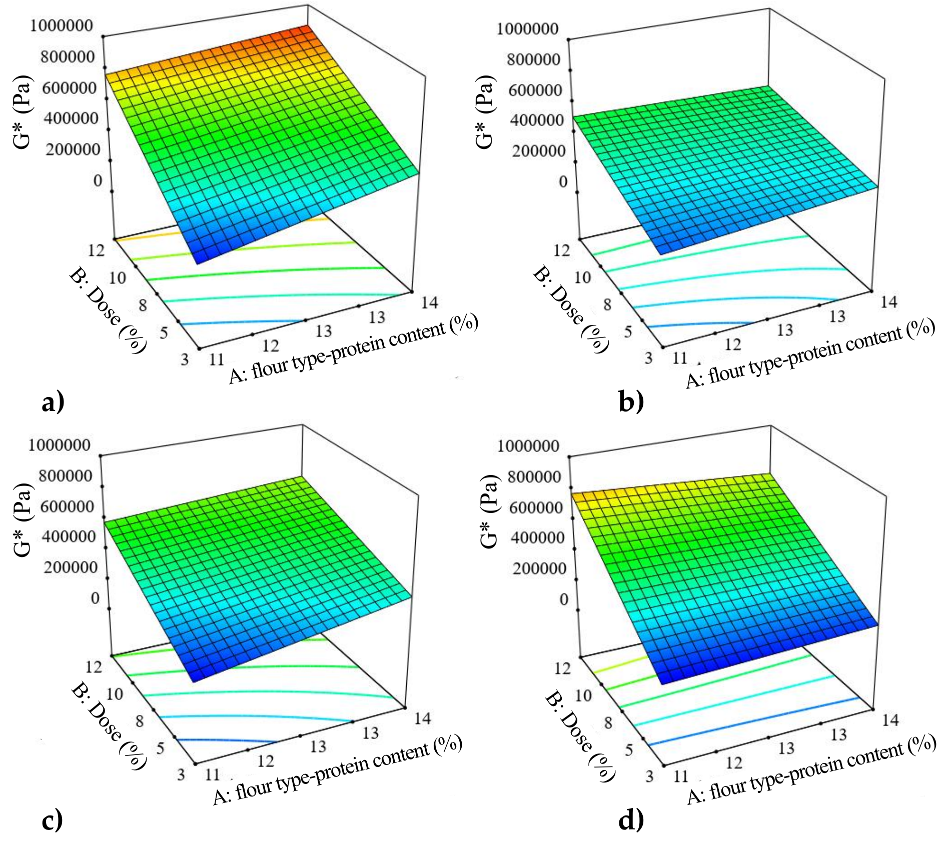


**Figure S.2.** The combined effect of flour type (protein content) and carrot pomace dosage from different varieties: a) Baltimore, b) Belgrado, c) Niagara, d) Sirkana on the complex modulus of dough (G*)

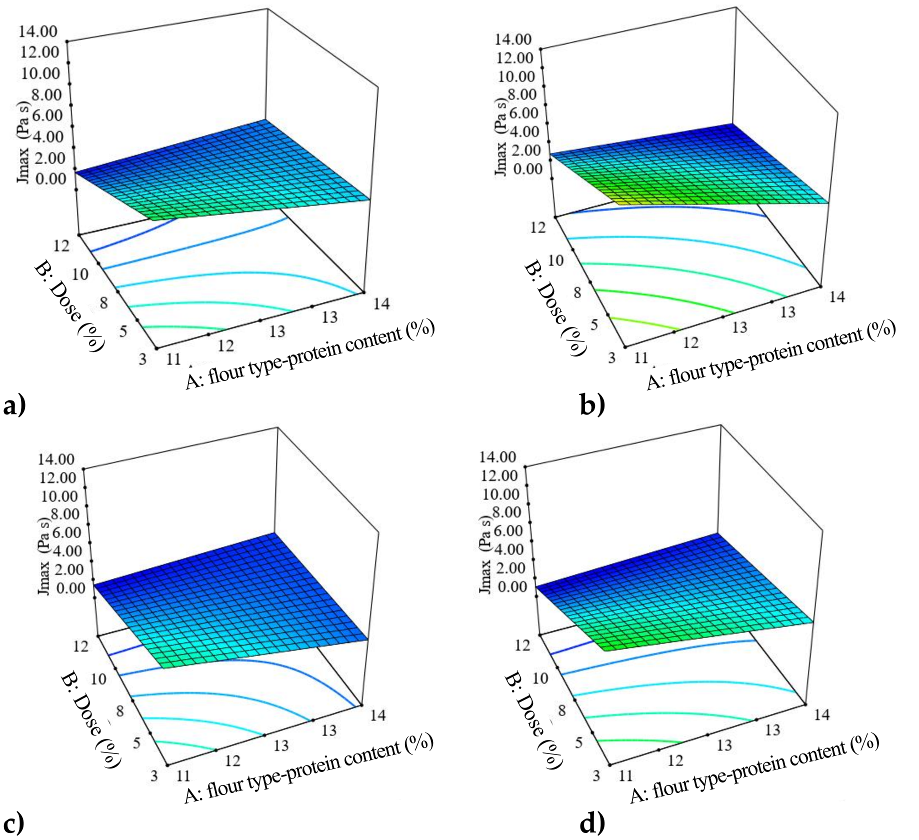


**Figure S.3.** Combined effect of flour type (protein content) and carrot pomace dosage of different varieties: a) Baltimore, b) Belgrado, c) Niagara, d) Sirkana on maximum dough compliance (Jmax)

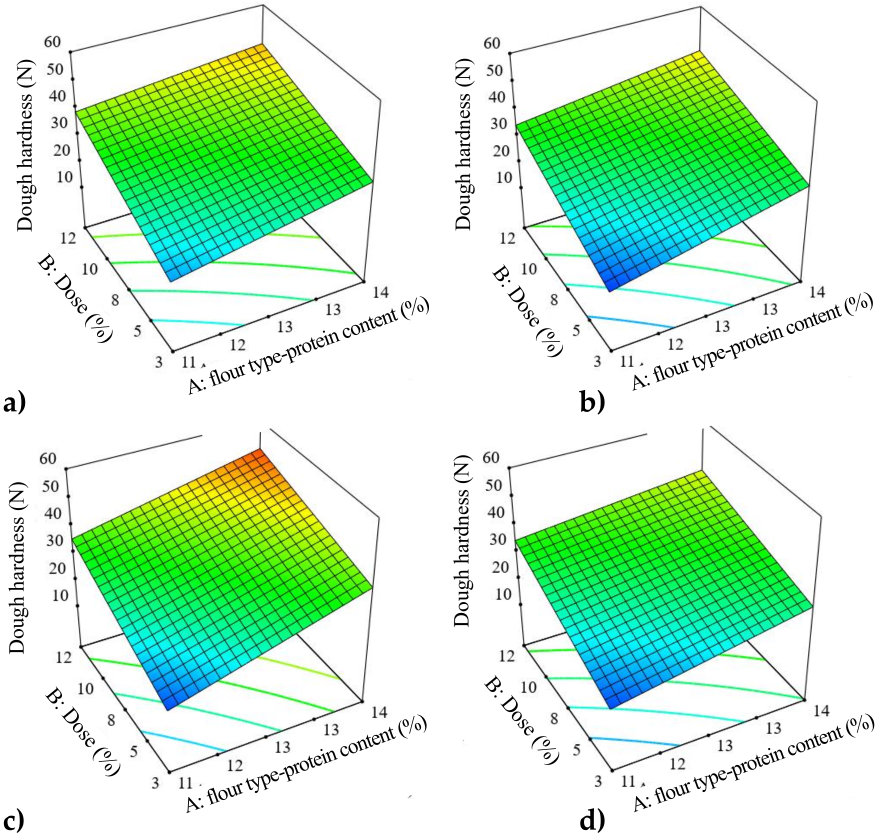


**Figure S.4.** The combined effect of flour type (protein content) and carrot pomace dosage from different varieties: a) Baltimore, b) Belgrado, c) Niagara, d) Sirkana on dough hardness

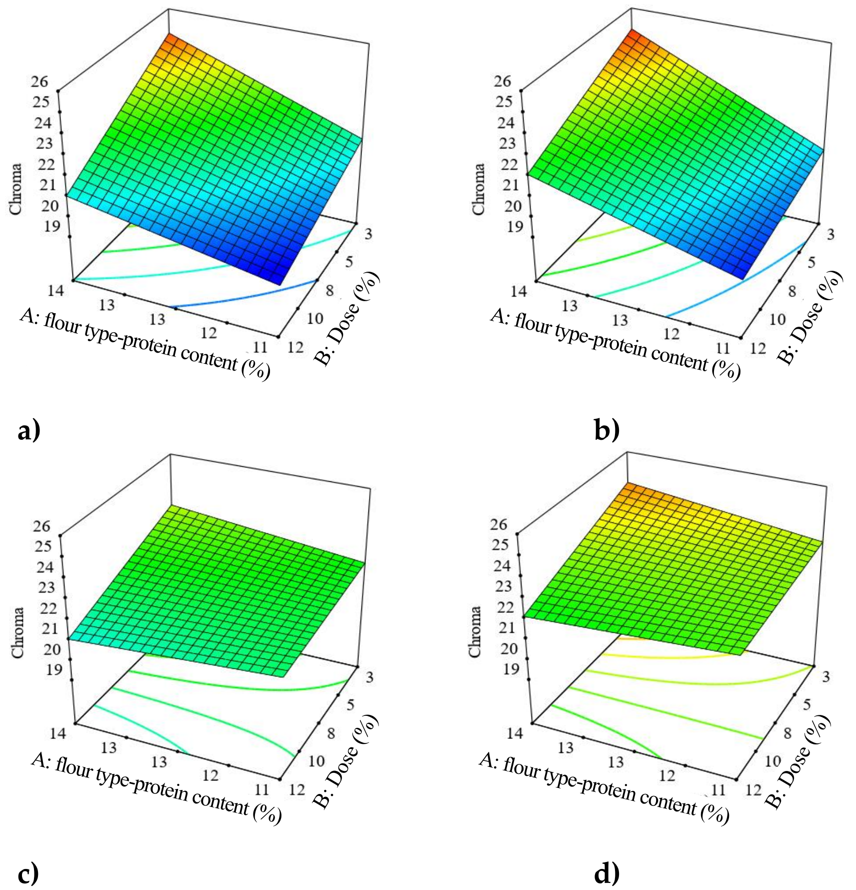


**Figure S.5.** The combined effect of flour type (protein content) and carrot pomace dosage from different varieties: a) Baltimore, b) Belgrado, c) Niagara, d) Sirkana on the color (Chroma) of pasta

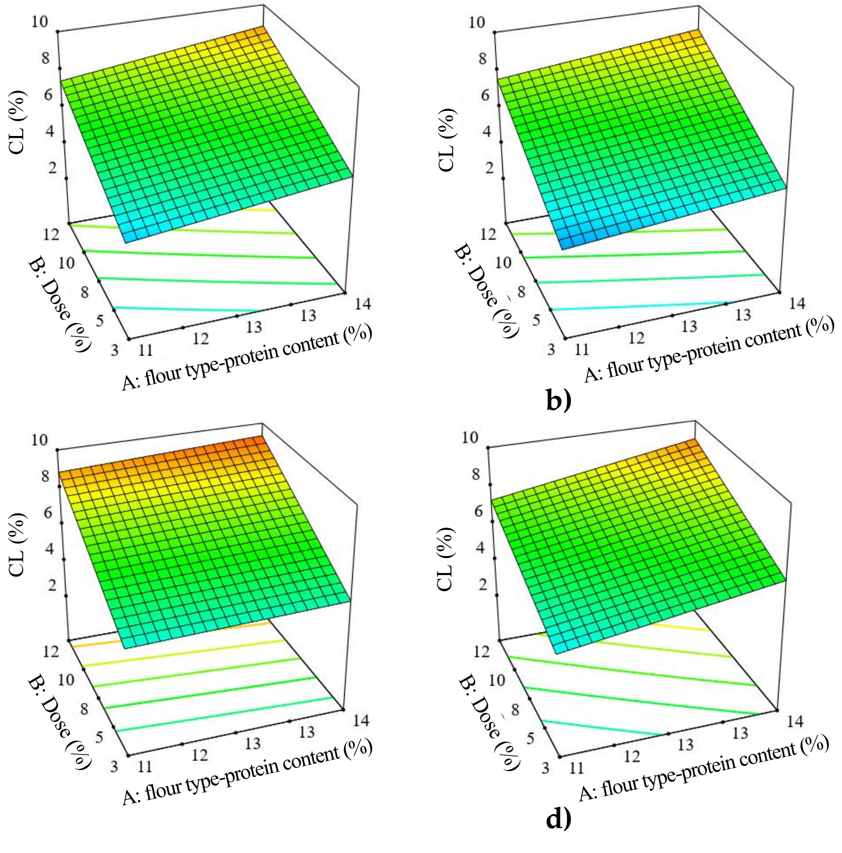


**Figure S.6.** The combined effect of flour type (protein content) and carrot pomace dosage from different varieties: a) Baltimore, b) Belgrado, c) Niagara, d) Sirkana on the loss of soluble solids (CL) of pasta

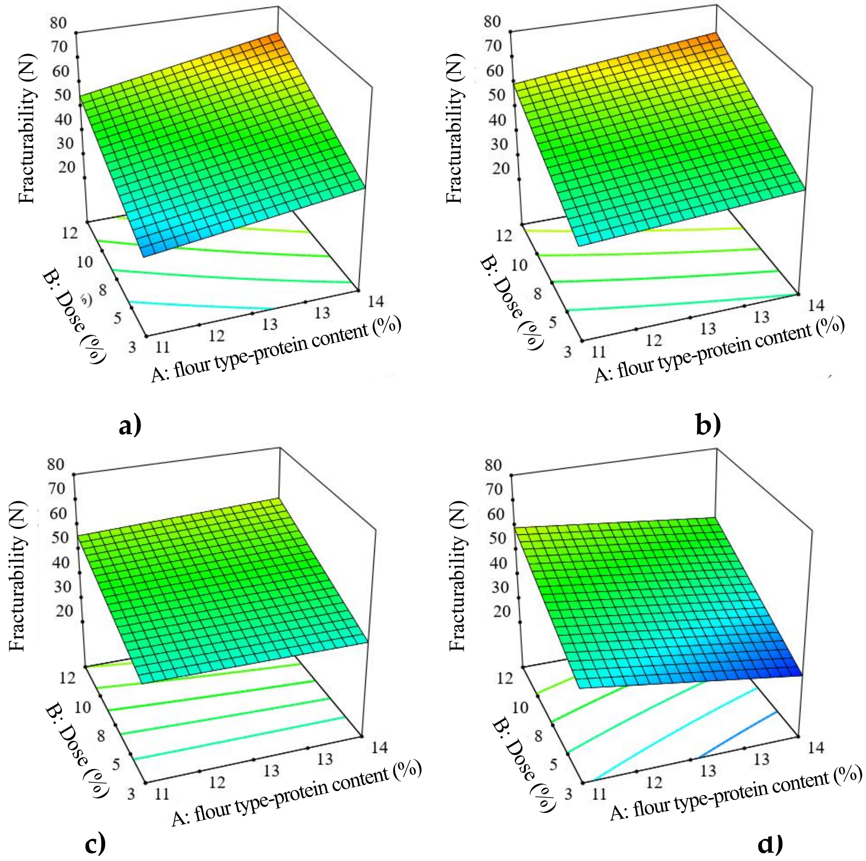


**Figure S.7.** The combined effect of flour type (protein content) and carrot pomace dosage from different varieties: a) Baltimore, b) Belgrado, c) Niagara, d) Sirkana on the fracturability of pasta

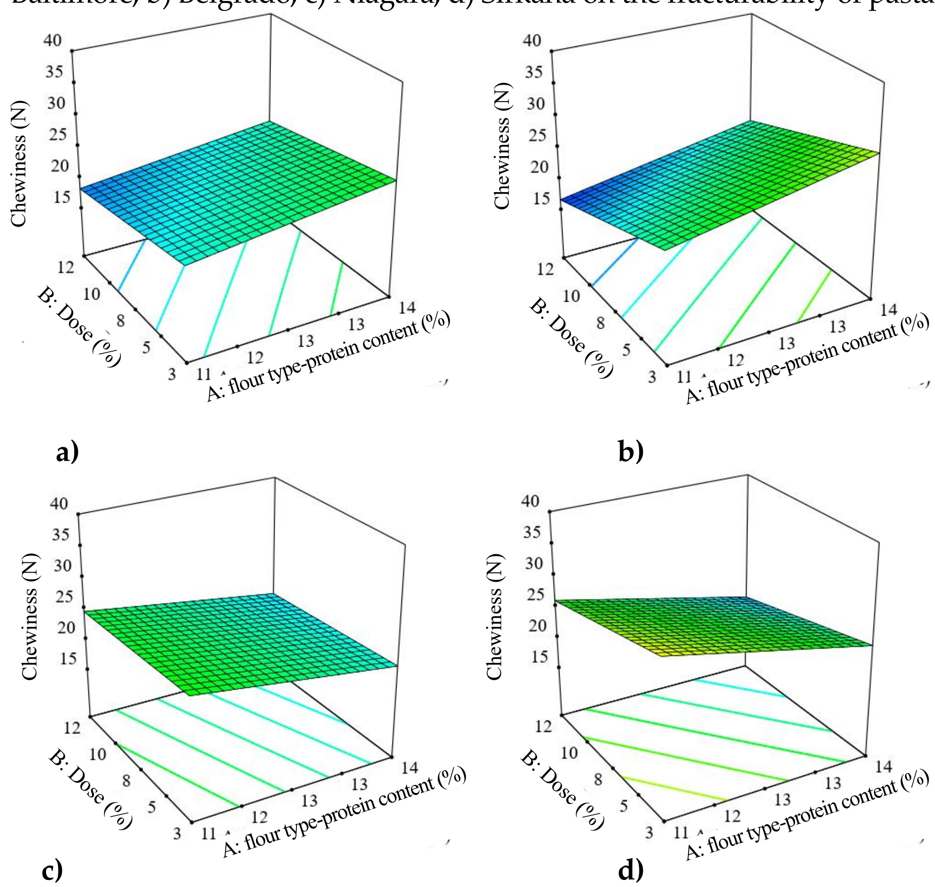


**Figure S.8.** The combined effect of flour type (protein content) and carrot pomace dosage from different varieties: a) Baltimore, b) Belgrado, c) Niagara, d) Sirkana on the chewiness of pasta

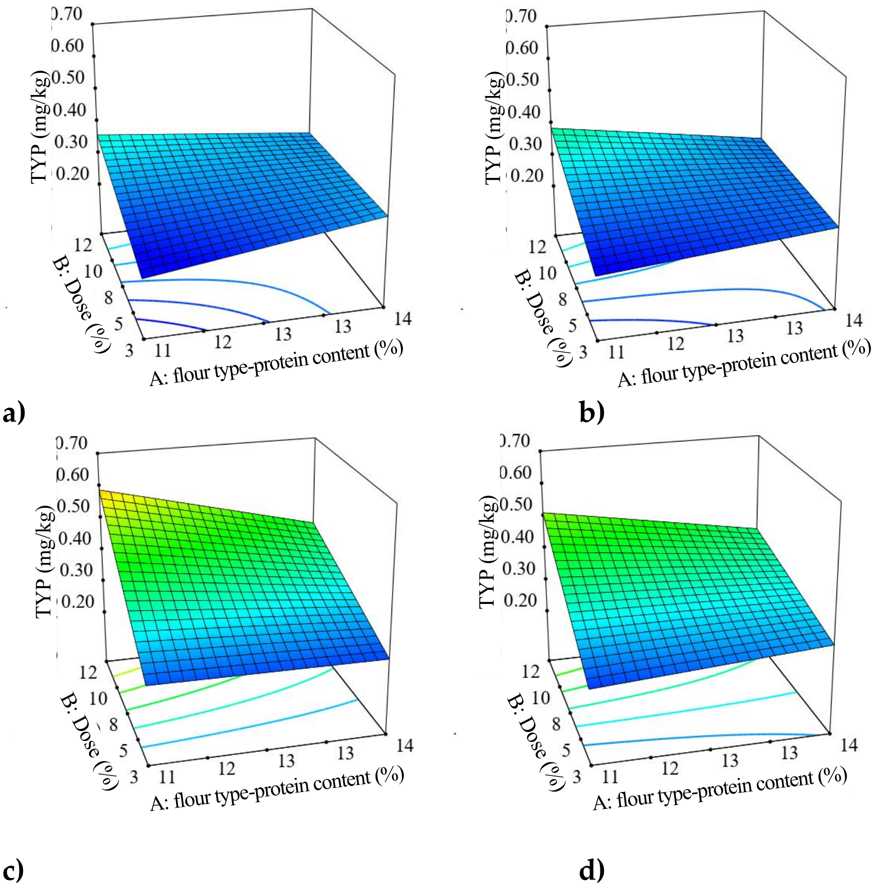


**Figure S.9.** The combined effect of flour type (protein content) and carrot pomace dosage from different varieties: a) Baltimore, b) Belgrado, c) Niagara, d) Sirkana on the total yellow pigment content of pasta

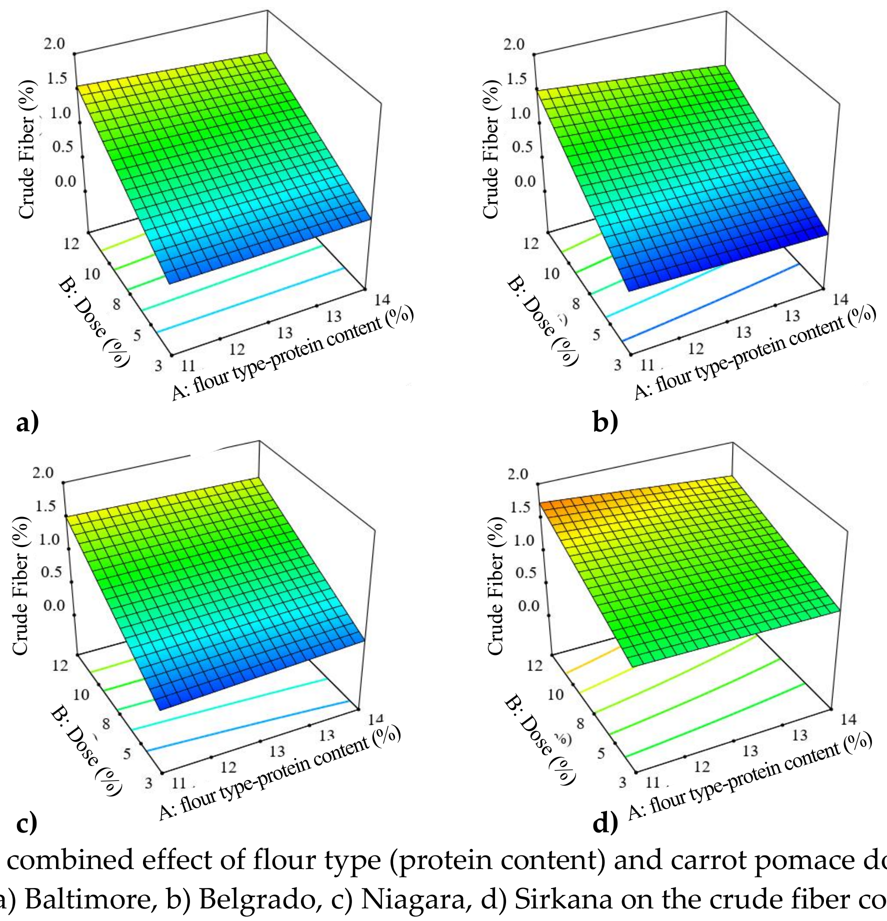


**Figure S.10.** The combined effect of flour type (protein content) and carrot pomace dosage from different varieties: a) Baltimore, b) Belgrado, c) Niagara, d) Sirkana on the crude fiber content of pasta

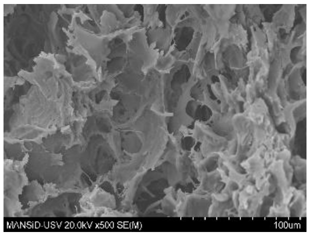


**F1**

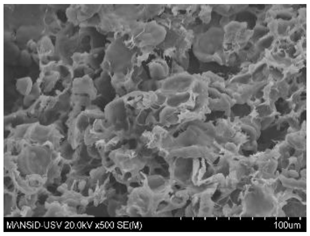


**F1Ba**

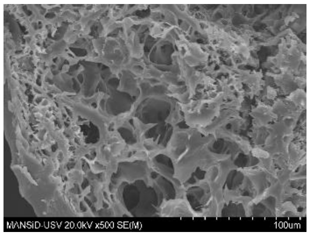


**F1Be**

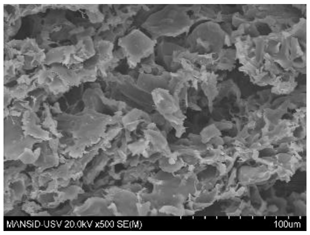


**F1Ni**

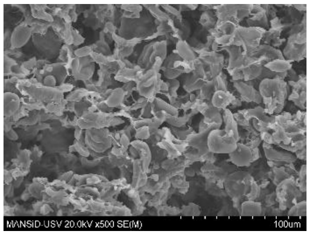


**F1Si**

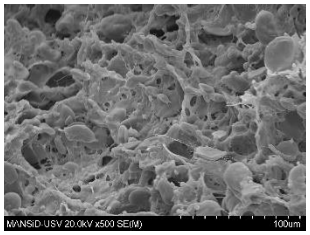


**F2**

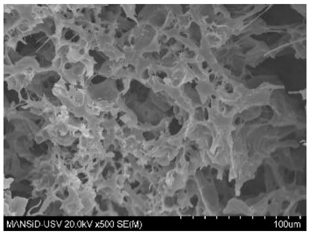


**F2Ba**

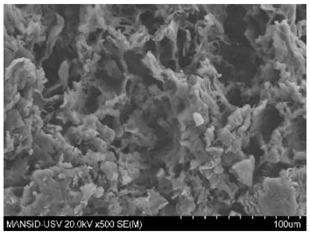


**F2Be**

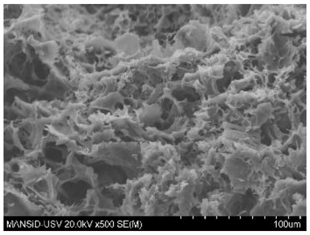


**F2Ni**

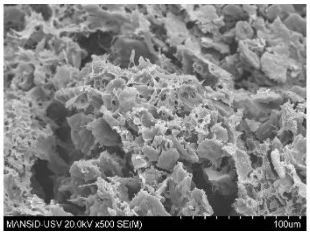


**F2Si**

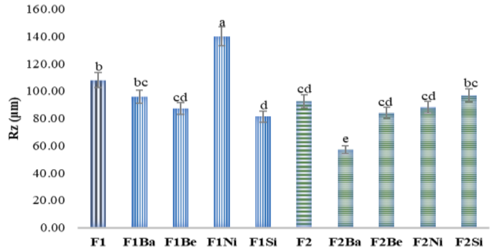


**Figure S.11.** The microstructure of cooked pasta and surface roughness (Rz) of uncooked pasta

**Table S2.** Correlations between flour and dough characteristics

| Variable | $G'_{10Hz}$ | $G''_{10Hz}$ | Dough hardness | $J_{max}$ | $G^*_{10Hz}$ | CH |
|---|---|---|---|---|---|---|
| $G'_{10Hz}$ | **1.00** | | | | | |
| $G''_{10Hz}$ | **0.99** | **1.00** | | | | |
| Dough hardness | **0.73** | **0.75** | **1.00** | | | |
| $J_{max}$ | **-0.69** | **-0.66** | -0.55 | **1.00** | | |
| $G^*_{10 Hz}$ | **0.85** | **0.84** | 0.53 | **-0.78** | **1.00** | |
| HC | **0.65** | **0.67** | **0.88** | -0.54 | 0.54 | **1.00** |

Bold values are significant at $p < 0.05$. G'10Hz – modulus of elasticity at a frequency of 10 Hz. G"10Hz – modulus of viscosity at a frequency of 10 Hz. Jmax – maximum compliance. G*10Hz complex modulus of dough at 10 Hz. HC– hydration capacity of flour.

**Table S3.** Correlations between the characteristics of the final product

| Variable | Zn | Fe | Cu | Na | OCT | WA | CL | L* | a* | b* | H | Appearance and form | Color | Smell | Taste | Flavor | Texture | General acceptability | TYP | Fracturability | Protein | Ash | Fiber | Carbohydrate | Energetic value | Chewability | Cohesiveness | Gumminess | Firmness | TPC |
|---|---|---|---|---|---|---|---|---|---|---|---|---|---|---|---|---|---|---|---|---|---|---|---|---|---|---|---|---|---|---|
| Zn | **1.00** | | | | | | | | | | | | | | | | | | | | | | | | | | | | | |
| Fe | **0.89** | **1.00** | | | | | | | | | | | | | | | | | | | | | | | | | | | | |
| Cu | **0.72** | **0.69** | **1.00** | | | | | | | | | | | | | | | | | | | | | | | | | | | |
| Na | 0.08 | 0.45 | 0.43 | **1.00** | | | | | | | | | | | | | | | | | | | | | | | | | | |
| OCT | -0.45 | -0.46 | -0.47 | -0.47 | **1.00** | | | | | | | | | | | | | | | | | | | | | | | | | |
| WA | -0.27 | -0.51 | -0.32 | -0.40 | 0.35 | **1.00** | | | | | | | | | | | | | | | | | | | | | | | | |
| CL | 0.04 | 0.28 | 0.51 | **0.78** | -0.54 | -0.54 | **1.00** | | | | | | | | | | | | | | | | | | | | | | | |
| L* | 0.38 | 0.13 | -0.01 | **-0.64** | 0.28 | 0.27 | **-0.64** | **1.00** | | | | | | | | | | | | | | | | | | | | | | |
| a* | -0.46 | -0.22 | 0.12 | 0.62 | -0.22 | -0.26 | **0.83** | **-0.83** | **1.00** | | | | | | | | | | | | | | | | | | | | | |
| b* | -0.23 | -0.07 | 0.36 | 0.62 | -0.29 | -0.19 | **0.89** | **-0.69** | **0.93** | **1.00** | | | | | | | | | | | | | | | | | | | | |
| H | 0.43 | 0.22 | -0.19 | -0.62 | 0.20 | 0.22 | **-0.81** | **0.86** | **-0.98** | **-0.94** | **1.00** | | | | | | | | | | | | | | | | | | | |
| Appearance and form | 0.13 | 0.34 | 0.61 | **0.72** | -0.51 | -0.62 | **0.90** | **-0.71** | **0.77** | **0.78** | **-0.79** | **1.00** | | | | | | | | | | | | | | | | | | |
| Color | 0.10 | 0.31 | 0.63 | **0.79** | -0.54 | -0.49 | **0.92** | **-0.74** | **0.80** | **0.84** | **-0.83** | **0.98** | **1.00** | | | | | | | | | | | | | | | | | |
| Smell | 0.09 | 0.28 | **0.64** | **0.78** | -0.55 | -0.46 | **0.94** | **-0.69** | **0.78** | **0.84** | **-0.81** | **0.95** | **0.98** | **1.00** | | | | | | | | | | | | | | | | |
| Taste | 0.04 | 0.20 | **0.65** | **0.65** | -0.34 | -0.42 | **0.90** | -0.60 | **0.81** | **0.90** | **-0.85** | **0.92** | **0.94** | **0.93** | **1.00** | | | | | | | | | | | | | | | |
| Flavor | -0.03 | 0.11 | 0.61 | **0.65** | -0.33 | -0.34 | **0.89** | -0.63 | **0.83** | **0.93** | **-0.87** | **0.87** | **0.92** | **0.93** | **0.98** | **1.00** | | | | | | | | | | | | | | |
| Texture | -0.20 | -0.03 | 0.46 | 0.55 | -0.13 | -0.35 | **0.86** | -0.61 | **0.87** | **0.93** | **-0.89** | **0.82** | **0.83** | **0.85** | **0.95** | **0.96** | **1.00** | | | | | | | | | | | | | |
| General acceptability | -0.05 | 0.11 | 0.56 | **0.65** | -0.38 | -0.39 | **0.92** | **-0.71** | **0.87** | **0.93** | **-0.90** | **0.93** | **0.95** | **0.96** | **0.97** | **0.98** | **0.95** | **1.00** | | | | | | | | | | | | |
| TYP | -0.27 | -0.05 | 0.12 | **0.67** | -0.41 | -0.04 | **0.77** | -0.58 | **0.74** | **0.78** | **-0.69** | 0.54 | 0.62 | **0.70** | 0.57 | 0.62 | 0.62 | **0.66** | **1.00** | | | | | | | | | | | |
| Fracturability | 0.01 | 0.27 | 0.44 | **0.80** | -0.61 | -0.50 | **0.84** | **-0.74** | **0.75** | **0.69** | **-0.74** | **0.91** | **0.92** | **0.90** | **0.77** | **0.73** | **0.66** | **0.82** | **0.66** | **1.00** | | | | | | | | | | |
| Protein | **0.92** | **0.91** | 0.54 | 0.14 | -0.25 | -0.29 | -0.08 | 0.38 | -0.56 | -0.39 | 0.54 | 0.00 | -0.04 | -0.04 | -0.13 | -0.20 | -0.32 | -0.21 | -0.30 | -0.07 | **1.00** | | | | | | | | | |
| Ash | 0.26 | 0.44 | 0.62 | **0.74** | **-0.71** | -0.53 | **0.90** | -0.50 | 0.60 | **0.67** | -0.58 | **0.86** | **0.86** | **0.91** | **0.77** | **0.74** | **0.66** | **0.80** | **0.73** | **0.88** | 0.15 | **1.00** | | | | | | | | |
| Fiber | -0.22 | 0.02 | 0.13 | **0.70** | -0.42 | -0.14 | **0.83** | **-0.67** | **0.82** | **0.86** | **-0.76** | 0.61 | **0.68** | **0.71** | **0.64** | **0.67** | **0.66** | **0.71** | **0.94** | **0.66** | -0.28 | **0.69** | **1.00** | | | | | | | |
| Carbohydrate | **-0.87** | **-0.95** | **-0.71** | -0.50 | 0.61 | 0.45 | -0.37 | -0.05 | 0.16 | -0.03 | -0.15 | -0.37 | -0.37 | -0.39 | -0.24 | -0.20 | -0.03 | -0.19 | -0.12 | -0.31 | **-0.87** | -0.56 | -0.15 | **1.00** | | | | | | |
| Energetic value | -0.16 | -0.35 | -0.49 | -0.56 | 0.17 | 0.26 | **-0.70** | 0.29 | -0.48 | -0.60 | 0.45 | **-0.63** | -0.62 | **-0.69** | **-0.65** | -0.61 | **-0.65** | **-0.66** | **-0.69** | -0.59 | -0.18 | **-0.76** | -0.63 | 0.39 | **1.00** | | | | | |
| Chewability | -0.13 | -0.01 | 0.41 | **0.68** | -0.47 | 0.00 | **0.73** | **-0.76** | **0.77** | **0.80** | **-0.82** | **0.75** | **0.84** | **0.86** | **0.75** | **0.79** | **0.70** | **0.83** | **0.77** | **0.82** | -0.26 | **0.73** | **0.73** | -0.13 | -0.57 | **1.00** | | | | |
| Cohesiveness | -0.20 | -0.24 | 0.33 | 0.37 | -0.27 | 0.31 | 0.49 | -0.62 | **0.66** | **0.75** | **-0.75** | 0.52 | **0.64** | **0.63** | **0.64** | **0.70** | 0.62 | **0.70** | 0.55 | 0.51 | -0.41 | 0.40 | 0.56 | 0.11 | -0.31 | **0.88** | **1.00** | | | |
| Gumminess | -0.11 | 0.01 | 0.43 | **0.69** | -0.46 | -0.02 | **0.73** | **-0.78** | **0.76** | **0.80** | **-0.82** | **0.77** | **0.86** | **0.87** | **0.76** | **0.80** | **0.71** | **0.84** | **0.74** | **0.81** | -0.23 | **0.72** | **0.71** | -0.15 | -0.56 | **0.99** | **0.89** | **1.00** | | |
| Firmness | -0.04 | 0.25 | 0.33 | **0.90** | **-0.64** | -0.41 | **0.89** | **-0.79** | **0.78** | **0.75** | **-0.76** | **0.81** | **0.87** | **0.87** | **0.71** | **0.72** | **0.64** | **0.78** | **0.83** | **0.91** | -0.08 | **0.86** | **0.85** | -0.38 | -0.58 | **0.82** | 0.51 | **0.81** | **1.00** | |
| TPC | -0.15 | -0.23 | -0.60 | -0.45 | 0.35 | 0.56 | **-0.78** | 0.61 | **-0.64** | **-0.71** | **0.69** | **-0.90** | **-0.85** | **-0.86** | **-0.86** | **-0.84** | **-0.83** | **-0.90** | -0.41 | **-0.70** | -0.01 | **-0.75** | -0.46 | 0.31 | 0.60 | **-0.64** | -0.50 | **-0.67** | -0.61 | **1.00** |

Bold values are significant at $p < 0.05$; OCT – optimum cooking time. WA – water absorption. TPC – total polyphenol content. L* - brightness. a*- green-red hue. b*- blue-yellow hue. H – hue angle. TYP – total yellow pigments
